# Vaginal *Lactobacillus* fatty acid response mechanisms reveal a metabolite-targeted strategy for bacterial vaginosis treatment

**DOI:** 10.1016/j.cell.2024.07.029

**Published:** 2024-09-19

**Authors:** Meilin Zhu, Matthew W. Frank, Christopher D. Radka, Sarah Jeanfavre, Jiawu Xu, Megan W. Tse, Julian Avila Pacheco, Jae Sun Kim, Kerry Pierce, Amy Deik, Fatima Aysha Hussain, Joseph Elsherbini, Salina Hussain, Nondumiso Xulu, Nasreen Khan, Vanessa Pillay, Caroline M. Mitchell, Krista L. Dong, Thumbi Ndung'u, Clary B. Clish, Charles O. Rock, Paul C. Blainey, Seth M. Bloom, Douglas S. Kwon

**Affiliations:** 1 Department of Biological Engineering, Massachusetts Institute of Technology, Cambridge, MA, USA; 2 Broad Institute of MIT and Harvard, Cambridge, MA, USA; 3 Ragon Institute of MGH, MIT, and Harvard, Cambridge, MA, USA; 4 Department of Host-Microbe Interactions, St. Jude Children's Research Hospital, Memphis, TN, USA; 5 Department of Microbiology, Immunology, and Molecular Genetics, University of Kentucky, Lexington, KY, USA; 6 Harvard Medical School, Boston, MA, USA; 7 HIV Pathogenesis Programme, The Doris Duke Medical Research Institute, University of KwaZulu-Natal, Durban, South Africa; 8 Health Systems Trust, Durban, South Africa; 9 Department of Obstetrics and Gynecology, Massachusetts General Hospital, Boston, MA, USA; 10 Division of Infectious Diseases, Massachusetts General Hospital, Boston, MA, USA; 11 Africa Health Research Institute, Durban, South Africa; 12 Max Planck Institute for Infection Biology, Berlin, Germany; 13 Division of Infection and Immunity, University College London, London, UK; 14 Koch Institute for Integrative Cancer Research at Massachusetts Institute of Technology, Cambridge, MA, USA

**Keywords:** vaginal microbiome, bacterial vaginosis, metabolism, female genital tract, women’s health, *Lactobacillus*

## Abstract

Bacterial vaginosis (BV), a common syndrome characterized by *Lactobacillus*-deficient vaginal microbiota, is associated with adverse health outcomes. BV often recurs after standard antibiotic therapy in part because antibiotics promote microbiota dominance by *Lactobacillus iners* instead of *Lactobacillus crispatus*, which has more beneficial health associations. Strategies to promote *L. crispatus* and inhibit *L. iners* are thus needed. We show that oleic acid (OA) and similar long-chain fatty acids simultaneously inhibit *L. iners* and enhance *L. crispatus* growth. These phenotypes require OA-inducible genes conserved in *L. crispatus* and related lactobacilli, including an oleate hydratase (*ohyA*) and putative fatty acid efflux pump (*farE*). FarE mediates OA resistance, while OhyA is robustly active in the vaginal microbiota and enhances bacterial fitness by biochemically sequestering OA in a derivative form only *ohyA*-harboring organisms can exploit. OA promotes *L. crispatus* dominance more effectively than antibiotics in an *in vitro* BV model, suggesting a metabolite-based treatment approach.

## Highlights

• OA inhibits bacteria linked to BV and BV risk
• OA enhances growth of health-associated vaginal lactobacilli via conserved mechanisms
• *Lactobacillus* OA response genes are robustly active in the human vaginal microbiome
• OA and related metabolites may improve current BV therapies for cervicovaginal health

## Graphical abstract

**Figure:**
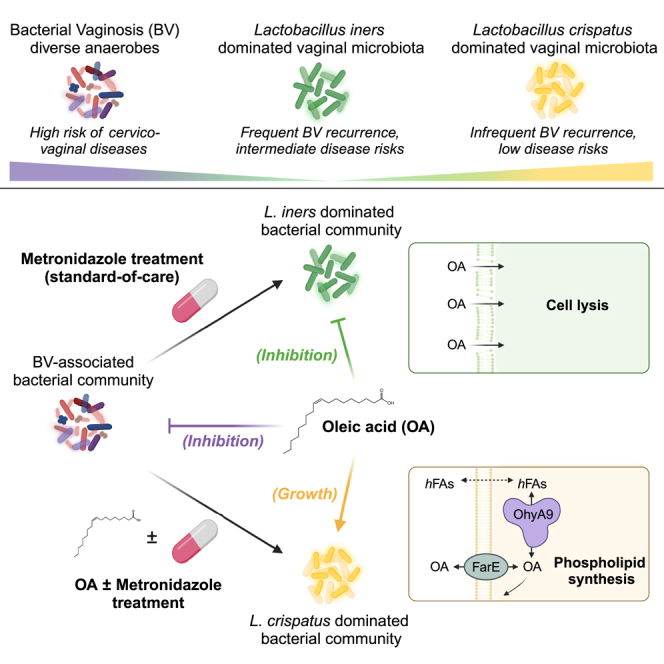

## Teaser

Vaginal microbiota dominance by *Lactobacillus iners* is associated with recurrence of bacterial vaginosis (BV), a widely prevalent syndrome, whereas microbiota dominance by other *Lactobacillus* species is more protective. Unsaturated long-chain fatty acids like oleic acid can simultaneously inhibit *L. iners* and BV-associated bacteria while promoting growth of non-*iners* lactobacilli, promoting a more health-associated microbiome composition in an *in vitro* model.

## Introduction

Specific female genital tract (FGT) microbiota communities are linked to adverse health outcomes such as preterm birth,^1^ infertility,^2^^,^^3^^,^^4^^,^^5^ cervical dysplasia,^6^^,^^7^^,^^8^ and sexually transmitted infections,^9^ including human immunodeficiency virus (HIV).^10^^,^^11^ Bacterial vaginosis (BV)—a syndrome of the FGT microbiota associated with these adverse outcomes—affects up to 58% of women worldwide.^12^ BV has clinical features including discharge, odor, and mucosal inflammation^13^^,^^14^^,^^15^ and is microbiologically characterized by a paucity of lactobacilli and predominance of diverse anaerobic bacteria.^16^^,^^17^ Conversely, health-associated FGT microbial communities are typically dominated by a single *Lactobacillus* species, most notably *Lactobacillus crispatus*, but also sometimes *Lactobacillus gasseri*, *Lactobacillus jensenii*, or *Lactobacillus mulieris*. By contrast, communities dominated by a different common *Lactobacillus* species—*Lactobacillus iners*—have several sub-optimal health associations,^6^^,^^10^^,^^18^ including higher risk of transitioning to BV.^19^^,^^20^^,^^21^

Standard first-line therapy with the antibiotic metronidazole (MTZ) resolves BV in some cases, but >50% of treated patients experience recurrence within 1 year.^22^^,^^23^^,^^24^ One potential explanation for frequent BV recurrence is MTZ’s tendency to shift FGT microbiota composition toward dominance by *L. iners*^21^^,^^25^^,^^26^^,^^27^^,^^28^ instead of *L. crispatus*. MTZ resistance among BV-associated (non-*Lactobacillus*) bacteria may also promote recurrence, although the clinical significance of MTZ resistance remains unclear.^29^^,^^30^^,^^31^ Investigational interventions employing adjunctive non-antibiotic strategies such as vaginal microbiota transplants and *L. crispatus*-containing live biotherapeutic products have shown promise compared with MTZ alone, but recurrence remains common.^24^^,^^32^^,^^33^ To our knowledge, no therapies currently exist to promote *L. crispatus* dominance in the FGT microbiota by selectively inhibiting *L. iners* or enhancing *L. crispatus* growth. However, *L. iners*’s reduced genome size and metabolic capacity relative to other FGT lactobacilli^34^^,^^35^ suggest feasibility of targeting metabolic differences to inhibit *L. iners* or promote *L. crispatus*.^36^

Mammalian mucosal surfaces are rich in long-chain fatty acids (LCFAs),^37^^,^^38^^,^^39^^,^^40^ which serve as critical nutrients and building blocks for bacterial membrane components and other processes.^41^ However, certain unsaturated LCFAs (uLCFAs) can also exert antimicrobial activity against gram-positive organisms like *Staphylococcus aureus*.^42^^,^^43^ The effects of uLCFAs on FGT *Lactobacillus* species have not been systematically assessed. We hypothesized that uLCFA metabolism might constitute a target to differentially modulate FGT lactobacilli.

Here, we investigated effects of *cis-*9-uLCFAs such as oleic acid (OA) on FGT *Lactobacillus* species and assessed their potential to modulate microbiota composition. We found that *cis-*9-uLCFAs selectively inhibited *L. iners* but robustly promoted non-*iners* FGT *Lactobacillus* growth. Using transcriptomic and genomic analyses, we identified OA-upregulated genes, including a putative fatty acid efflux pump (*farE*) and oleate hydratase (*ohyA*), that were conserved in non-*iners* FGT *Lactobacillus* species but absent in *L. iners*, mirroring the observed growth phenotypes. Using a genetically tractable *L. gasseri* strain, we showed *farE* is required for OA resistance. Characterization of *Lactobacillus* OhyA enzymes showed they are robustly active *in vivo* and revealed that non-*iners* FGT lactobacilli can utilize OhyA to biochemically sequester OA for use in phospholipid synthesis. Overall, these data support that *ohyA* and *farE* confer non-*iners* lactobacilli a competitive advantage in *cis*-9-uLCFA-rich environments. We leverage these discoveries to show that OA treatment alone or in combination with MTZ can promote *L. crispatus* dominance in BV-like bacterial communities *in vitro*. Collectively, this work describes important species-level differences in FGT *Lactobacillus* metabolism, elucidates mechanisms underlying those differences, and proposes a metabolism-targeted intervention to improve female genital and reproductive health.

## Results

### *cis*-9-uLCFAs selectively inhibit *L. iners* and enhance growth of *L. crispatus* and other FGT lactobacilli

**Figure 1:**
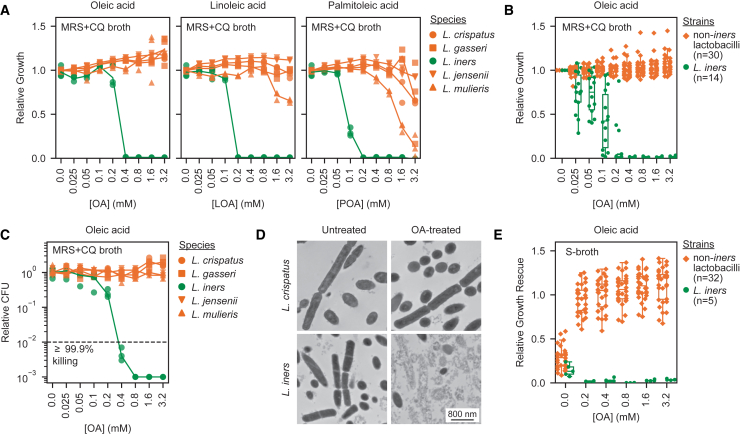
*cis*-9-uLCFAs selectively inhibit *L. iners* and promote growth of *L. crispatus* and other FGT lactobacilli (A) Growth of representative *L. crispatus*, *L. gasseri*, *L. iners*, *L. jensenii*, and *L. mulieris* strains in modified *Lactobacillus* MRS broth (MRS + CQ broth^36^) supplemented with varying concentrations of oleic acid (OA), linoleic acid (LOA), or palmitoleic acid (POA). Calculated as growth relative to the median of no-LCFA control cultures. (B) Relative growth of diverse non-*iners* FGT *Lactobacillus* (*n* = 30) and *L. iners* (*n* = 14) strains in MRS + CQ broth supplemented with varying OA concentrations. (C) Minimum bactericidal concentration (MBC) assays for representative *L. crispatus*, *L. gasseri*, *L. iners*, *L. jensenii*, and *L. mulieris* strains in MRS + CQ broth. Colony forming units (CFUs) were measured after 24 h of OA exposure and expressed relative to CFU from no-OA controls. (D) Transmission electron microscopy (TEM) images of *L. crispatus* (top) and *L. iners* (bottom) treated with 3.2 mM OA (right) or no OA (left) for 1 h. (E) Growth rescue of non-*iners* FGT *Lactobacillus* (*n* = 32) and *L. iners* (*n* = 5) strains in S-broth^36^ supplemented with varying OA concentrations. Calculated relative to growth in S-broth supplemented with 0.1% (v/v) Tween-80. (A, B, and E) Growth was measured by optical density at 600 nm (OD600) after 72 h. (A and C) Points represent 3 technical replicates per condition and are representative of ≥2 independent experiments. (B and E) Points represent median values for 3 technical replicates per condition and are representative of ≥2 independent experiments. Boxplots represent the 25^th^ and 75^th^ percentiles (lower and upper boundaries of boxes, respectively), the median (middle horizontal line), and measurements that fall within 1.5 times the interquartile range (IQR; distance between the 25^th^ and 75^th^ percentiles; whiskers). See also Figure S1.

**Figure S1:**
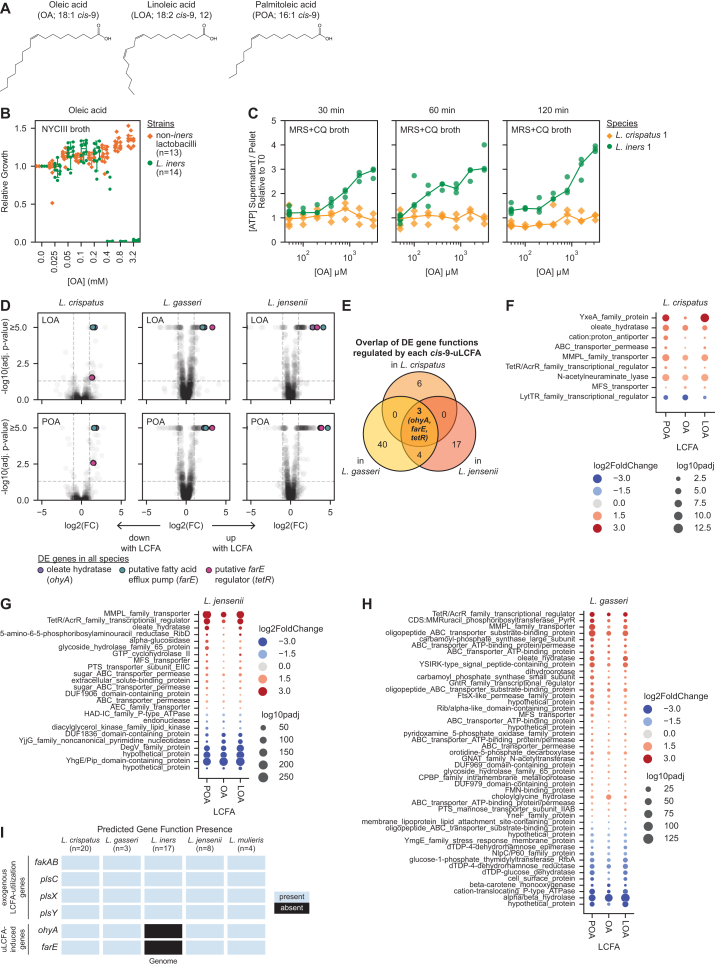
*cis*-9-uLCFA structures, FGT *Lactobacillus cis*-9-uLCFA responses, and *cis*-9-uLCFA response gene presence in FGT *Lactobacillus* species, related to Figures 1 and 2 (A) Chemical structures of oleic acid, linoleic acid, and palmitoleic acid. (B) Growth of diverse non-*iners* FGT *Lactobacillus* (*n* = 13 strains) and *L. iners* (*n* = 14 strains) in NYCIII broth supplemented with varying concentrations of OA. Growth was measured by OD600 after 72 h of culture. Relative growth for each strain was calculated relative to the median OD600 measurement in its no-LCFA control. Points represent median relative growth for 3 technical replicates per condition. Boxplots represent the 25^th^ and 75^th^ percentiles (lower and upper boundaries of boxes, respectively), the median (middle horizontal line), and measurements that fall within 1.5 times the IQR (whiskers). (C) Cultures of *L. iners* and *L. crispatus* were grown to mid/late-log phase, and then parallel aliquots of each strain were exposed to varying concentrations of OA for the indicated amount of time, followed by centrifugation and separation of supernatants and pellets. ATP concentrations were determined for each supernatant and pellet. ATP release was calculated by dividing the supernatant ATP concentration by the pellet ATP concentration and expressed relative to the strains ATP release at T0. (D) Transcriptional responses of cultured *L. crispatus* (left), *L. gasseri* (middle), and *L. jensenii* (right) grown to exponential phase in MRS + CQ broth, then exposed to LOA (top, 3.2 mM) or POA (bottom, 3.2 mM) for 1 h. Data were analyzed as in Figure 2A. Consistently, DE genes included a predicted *ohyA*, putative *farE*, and its putative *tetR*. The untreated (no-uLCFA) controls for each species in Figures 2A and S1D are the same. (E) Venn diagram showing the shared sets of genes differentially expressed in response to each of the three *cis*-9-uLCFAs (OA, LOA, and POA) in each species and the overlap between these sets of DE gene functions that were shared among all three species. Three shared DE gene functions (*ohyA*, *farE*, and *tetR*; all upregulated) were observed in all species under all treatment conditions. (F–H) Dot plot showing the shared sets of DE genes induced by OA, LOA, and POA treatments in *L. crispatus* (F), *L. gasseri* (G), and *L. jensenii* (H). Each point depicts a DE gene with color representing log_2_(FC) and size representing −log_10_(adjusted *p* value). (I) Presence of gene functions predicted to encode oleate hydratase (*ohyA*) and putative fatty acid efflux pump (*farE*) activity in long-read sequenced, isolate genomes of strains from the indicated FGT *Lactobacillus* species, representing all *Lactobacillus* strains used experimentally in this study (see also Table S1). Presence of gene functions involved in exogenous fatty acid acquisition and utilization (*fakAB*, *plsC*, *plsX*, and *plsY*) is shown for comparison.

To investigate *cis-*9-uLCFA effects on FGT *Lactobacillus* species, we cultured representative strains with *cis-*9-uLCFAs in modified *Lactobacillus* MRS broth (MRS + CQ broth; De Man, Rogosa, and Sharpe broth with cysteine and glutamine).^36^ Strikingly, OA (18:1 *cis-*9), linoleic acid (LOA; 18:2 *cis-*9,12), and palmitoleic acid (POA; 16:1 *cis-*9) each potently inhibited *L. iners* while exerting little or no inhibitory effect on *L. crispatus*, *L. gasseri*, *L. jensenii*, and *L. mulieris* (Figure 1A; chemical structures shown in Figure S1A). OA inhibited *L. iners* at a half-maximal inhibitory concentration (IC50) of <400 μM, but none of the non-*iners* species were inhibited at concentrations up to 3.2 mM. To assess generalizability of these findings, we tested diverse strains from each species, including isolates from geographically diverse donors with varying BV status^36^ (Table S1). All *L. iners* strains (*n* = 14) were OA-susceptible (median IC50 of 100 μM in MRS + CQ broth), while non-*iners* FGT *Lactobacillus* strains (*n* = 30) were not inhibited (Figure 1B). Similar results were observed in New York City III broth (NYCIII broth), a rich, non-selective media commonly used to culture FGT bacteria^44^ (Figure S1B).

To evaluate whether OA inhibition of *L. iners* was bactericidal, we performed minimum bactericidal concentration (MBC) assays^45^ using representative *Lactobacillus* strains in MRS + CQ broth. The minimum OA concentration required to kill ≥99.9% of the *L. iners* inoculum within 24 h (the MBC) was 400 μM, matching the strain’s corresponding minimum concentration (MIC achieving ≥99.9% growth inhibition; Figures 1A and 1C), indicating a bactericidal effect. By contrast, OA failed to kill the non-*iners Lactobacillus* species. To assess whether OA killed *L. iners* via membrane disruption (as reported for uLCFA-driven *S. aureus* inhibition^42^), we measured release of intracellular ATP. OA treatment of *L. iners* rapidly induced ATP release in dose- and time-dependent fashion at concentrations consistent with the MIC and MBC (Figure S1C), but did not induce ATP release from *L. crispatus*. Transmission electron microscopy confirmed high OA concentrations caused catastrophic cell wall and membrane disruption in *L. iners* by 1 h, whereas *L. crispatus* cell integrity and morphology remained unaffected (Figure 1D).

We next investigated effects of OA on growth of non-*iners* FGT *Lactobacillus* species using S-broth, a rich media formulation in which *Lactobacillus* species grow poorly without Tween-80.^36^ Supplementing S-broth with OA instead of Tween-80 robustly enhanced non-*iners* FGT *Lactobacillus* species growth (*n* = 32 strains) while inhibiting *L. iners* (*n* = 5 strains; Figure 1E).

### Non-*iners* FGT lactobacilli possess a conserved set of *cis*-9-uLCFA-response genes that *L. iners* lacks

**Figure 2:**
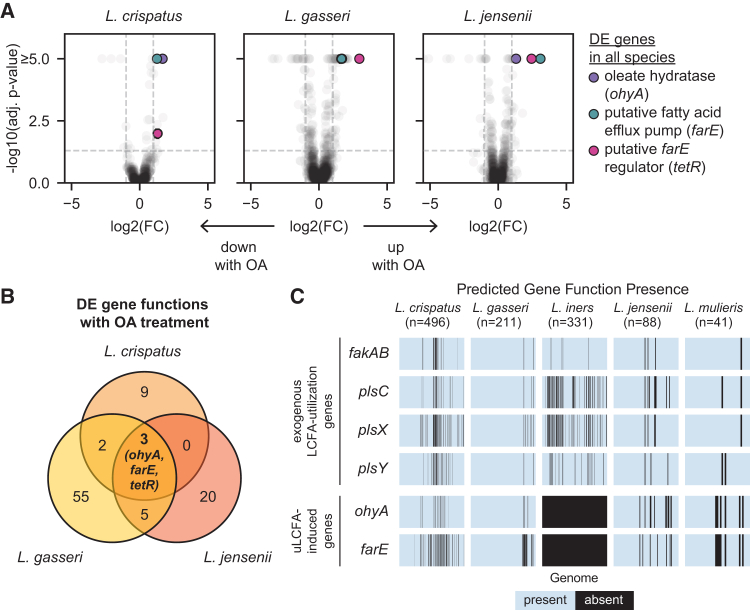
Non-*iners* FGT lactobacilli share a conserved set of OA response genes that *L. iners* lacks (A) Transcriptional responses of cultured *L. crispatus* (left), *L. gasseri* (middle), and *L. jensenii* (right) to OA (3.2 mM). Plots depict log_2_(fold change [FC]) in OA relative to control and −log_10_(adjusted *p* value) for each gene. Dotted lines indicate significant differential expression (FC ≥ −1 or FC ≥ 1 with adjusted *p* ≤ 0.05). (B) Venn diagram showing numbers of OA-regulated genes in each species and shared among species. Three gene functions (*ohyA*, COG4716; *farE*, COG2409; and *tetR*, COG1309) were consistently OA-regulated. (C) Presence of predicted *ohyA* and *farE* genes in isolate and metagenome-assembled genomes (MAGs) of FGT *Lactobacillus* species (*n* = 1,167 total).^36^^,^^47^ Predicted genes involved in exogenous fatty acid acquisition and utilization (*fakAB*, *plsC*, *plsX*, and *plsY*) are shown for comparison. See also Figures S1, S2, and S3.

We hypothesized that non-*iners* FGT *Lactobacillus* species’ similar uLCFA growth responses reflected a shared response mechanism. We therefore assessed *L. crispatus*, *L. gasseri*, and *L. jensenii* transcriptional responses to OA. Representative strains were grown to exponential phase, then treated with OA for 1 h. Bulk RNA sequencing and DESeq2^46^ analysis identified genes differentially expressed (DE) in each species compared with untreated controls. Three DE gene functions (all upregulated) were common among all three species in response to OA (Figures 2A and 2B), including a predicted oleate hydratase (*ohyA*), a putative fatty acid efflux pump (*farE*), and its putative regulator (*tetR*). Transcriptomic analysis of the same strains treated with POA or LOA revealed upregulation of the same three gene functions (Figures S1D–S1H), confirming that these species shared a core set of *cis*-9-uLCFA response genes.

Given its strikingly different OA response, we hypothesized that *L. iners* lacked these core response genes shared by non-*iners* lactobacilli. Using a previously reported FGT *Lactobacillus* genome catalog comprising 1,161 isolate genomes and metagenome-assembled genomes (MAGs) and six separately published, completed *L. iners* isolate genomes, we confirmed that *ohyA* and *farE* gene functions were completely absent from *L. iners* but highly conserved among non-*iners* species (Figure 2C). *tetR*, *farE’s* putative regulator, was excluded from this analysis due to its nonspecific functional annotation. By contrast, genes involved in acquiring (*fakA* and *fakB*) and utilizing (*plsC*, *plsX*, and *plsY*) exogenous FAs for phospholipid synthesis were present in all species (Figure 2C),^48^ showing *L. iners* retains other FA-related pathways. We additionally long-read sequenced genomes of all *Lactobacillus* strains experimentally tested in this study, confirming these findings (Figure S1I). These results suggested *ohyA* and *farE* might mediate species-specific uLCFA growth phenotypes in FGT lactobacilli.

### Comparative genomics reveals conserved phylogeny of FGT *Lactobacillus ohyA* and *farE*

**Figure S2:**
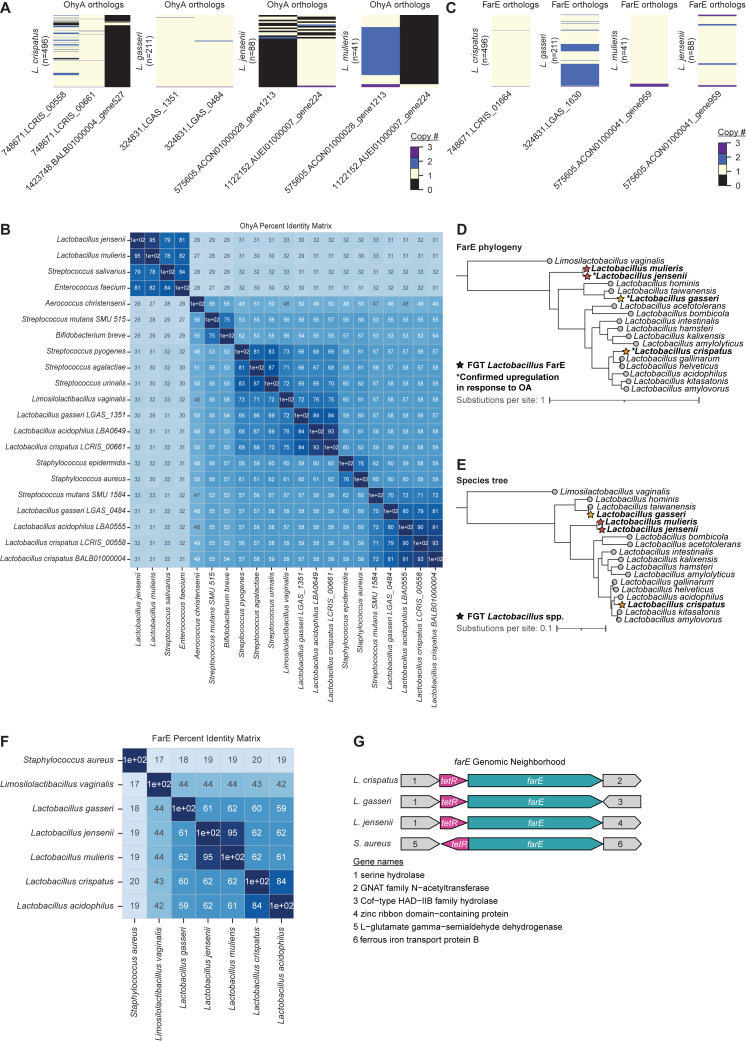
OhyA and FarE ortholog diversity in FGT *Lactobacillus* species and homology to orthologs found in other human-adapted species, related to Figures 2 and 3 (A) EggNOG-predicted orthologs for OhyA and the number of copies of each ortholog per genome or MAG for each species. Numbers of genomes and MAGs for each species are shown. (B) Percent identity matrix of representative OhyA orthologs in FGT *Lactobacillus* species and other human-adapted bacteria, determined by protein sequence alignment (MUSCLE v5.1^108^). (C) EggNOG-predicted orthologs for FarE and the number of copies of each ortholog per genome or MAG for each species. Numbers of genomes and MAGs for each species are shown. (D) FarE protein phylogenetic tree for representative *farE* orthologs from the indicated *Lactobacillus* species. Starred leaf tips indicate FGT *Lactobacillus* orthologs; ^∗^ indicates confirmed OA-induced ortholog (Figure 2A). (E) Species phylogenetic tree of representative *Lactobacillus* genomes constructed based on core ribosomal protein sequences. Starred leaf tips indicate FGT *Lactobacillus* species. (F) Percent identity matrix of representative EggNOG-predicted FarE orthologs in FGT *Lactobacillus* species and other human-adapted bacteria, determined by protein sequence alignment (MUSCLE v5.1^108^). (G) Schematic of *farE* and *tetR* genomic neighborhoods in representative *L. crispatus*, *L. gasseri*, *L. jensenii*, and *S. aureus* genomes (not to scale). (D and E) Trees were rooted to the FarE ortholog (D) or core genome (E) of the representative genome for *Limosilactobacillus vaginalis* (see STAR Methods).

**Figure 3:**
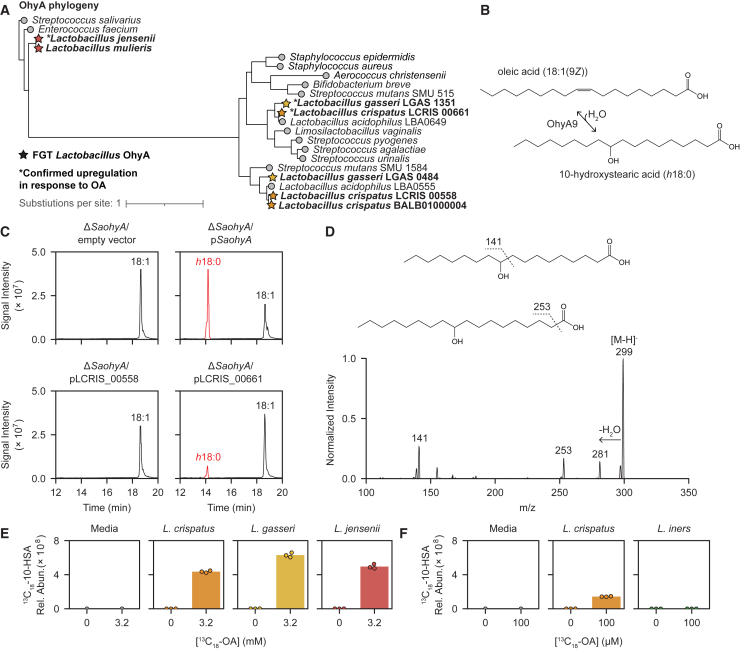
FGT *Lactobacillus* OhyA enzymes are functional and physiologically active (A) OhyA protein phylogenetic tree for representative orthologs from the indicated species (see STAR Methods). Starred leaf tips indicate FGT *Lactobacillus* orthologs; ^∗^ indicates confirmed OA-induced ortholog (Figure 2A). (B) Diagram of OhyA9 enzymatic activity with OA substrate and 10-hydroxystearic acid (10-HSA or *h*18:0) product. (C) Extracted ion chromatograms from supernatants of *ohyA*-gene deleted *S. aureus*^49^ complemented with empty vector (*ΔSaohyA*/empty vector), *SaohyA*-expressing plasmid (*ΔSaohyA*/p*SaohyA*), LCRIS_00558-expressing plasmid (*ΔSaohyA*/pLCRIS_00558), or LCRIS_00661-expressing plasmid (*ΔSaohyA*/pLCRIS_00661), cultured with OA for 1 h. Annotated peaks include OA (18:1) and 10-HSA (*h*18:0). (D) MS2 spectra with major fragmentation labels for the 10-HSA (*h*18:0) peak from *ΔSaohyA*/pLCRIS_00661 cultured with OA (Figure 3C, lower right). (E) Universally ^13^C-labeled 10-HSA (^13^C_18_-10-HSA) concentrations in supernatants of *L. crispatus*, *L. gasseri*, and *L. jensenii* cultured for 72 h in NYCIII broth with or without universally ^13^C-labeled OA (^13^C_18_-OA; 3.2 mM). (F) ^13^C_18_-10-HSA concentrations in supernatants of *L. crispatus* and *L. iners* cultured for 72 h in NYCIII broth with or without ^13^C_18_-OA (100 μM, a sublethal concentration for *L. iners*). (E and F) The same no-OA controls for media and *L. crispatus* are shown in (E) and (F). Points represent 3 technical replicates per condition. See also Figures S2 and S3.

We next genomically characterized *ohyA* and *farE* in FGT lactobacilli. Most *L. crispatus* and *L. gasseri* genomes contained two distinct predicted *ohyA* orthologs (only one of which was induced by OA in each species), whereas *L. jensenii* and *L. mulieris* genomes each contained a single ortholog (Figure S2A). Phylogenetic reconstruction of OhyA protein sequences from FGT *Lactobacillus* species and other gram-positive bacteria revealed three major groupings (Figure 3A; percent identity matrix shown in Figure S2B). The *L. crispatus* and *L. gasseri* OA-inducible orthologs (LCRIS_00661 and LGAS_1351, respectively) clustered with previously characterized orthologs from *S. aureus*,^49^^,^^50^ *Bifidobacterium breve*,^51^ *S. pyogenes*,^52^ and *Lactobacillus acidophilus*,^53^^,^^54^ whereas non-OA-induced *L. crispatus* and *L. gasseri* orthologs (LCRIS_00558/BALB01000004 and LGAS_0484) formed a separate cluster. *L. jensenii* and *L. mulieris* orthologs formed a more distant grouping with *Streptococcus salivarius* and *Enterococcus faecium* orthologs. In contrast to OhyA, non-*iners* FGT *Lactobacillus* species each contained a single *farE* ortholog, with phylogeny mirroring underlying species phylogeny (Figures S2C–S2E). *Lactobacillus* FarE shared distant homology (18%–20% amino acid identity) to the *S. aureus* FarE and similarly, genomically neighbored a *tetR* family regulator (Figures S2F and S2G). Thus, phylogeny of FGT *Lactobacillus ohyA* orthologs differs substantially from underlying species phylogeny, suggesting a complex evolutionary history involving horizontal gene transfer and/or species-specific gene loss, whereas *farE* phylogeny in *Lactobacillus* is consistent with vertical inheritance.

**Figure S3:**
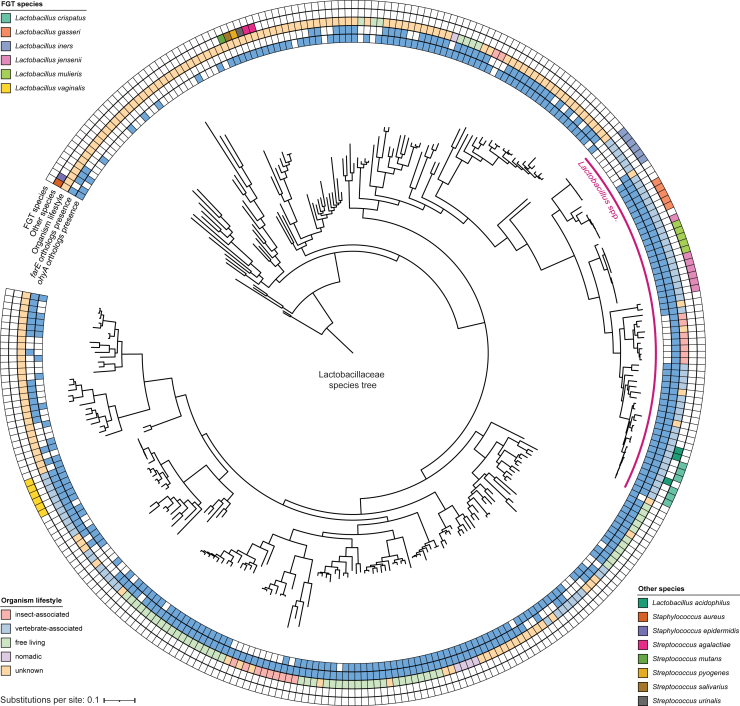
*ohyA* and *farE* orthologs presence is widespread among Lactobacillaceae species, related to Figures 2 and 3 Species phylogenetic tree of representative species genomes from the Lactobacillaceae family, constructed based on core ribosomal genes contained in all species. The species genomes derive from a recent comprehensive review and taxonomic revision of the 48 genera within the Lactobacillaceae family.^55^*Staphylococcus aureus* and *Staphylococcus epidermidis* genomes were included to root the phylogenetic reconstruction. Metadata rings mark the genomes of major FGT *Lactobacillus* species, genomes of other gram-positive host-adapted species, each organism’s lifestyle (if known), and presence or absence of *farE* and *ohyA* orthologs in each genome. With the exception of *L. iners*, all vertebrate-associated members of the *Lactobacillus* genus—including common mammalian intestinal *Lactobacillus* species (which also require the OA-containing media supplement Tween-80 for growth)—possessed both putative *ohyA* and *farE* genes. Trees were constructed from MUSCLE v5.1^108^-aligned protein sequences using FastTree v2.1 (see STAR Methods).

We next more broadly investigated *farE* and *ohyA* gene presence within the Lactobacillaceae family, including species with diverse lifestyles (e.g., vertebrate-associated, insect-associated, free-living, or nomadic).^55^ Both genes were widely (although not universally) distributed among Lactobacillaceae and often co-occurred (Figure S3). Notably, all *Lactobacillus* genus members except *L. iners* possessed *farE*, and all vertebrate-associated *Lactobacillus* species except *L. iners* possessed *ohyA*, suggesting an important conserved role of *cis*-9-uLCFA responses in environments beyond the FGT.

### FGT *Lactobacillus* OhyA enzymes are functional and physiologically active

**Figure S4:**
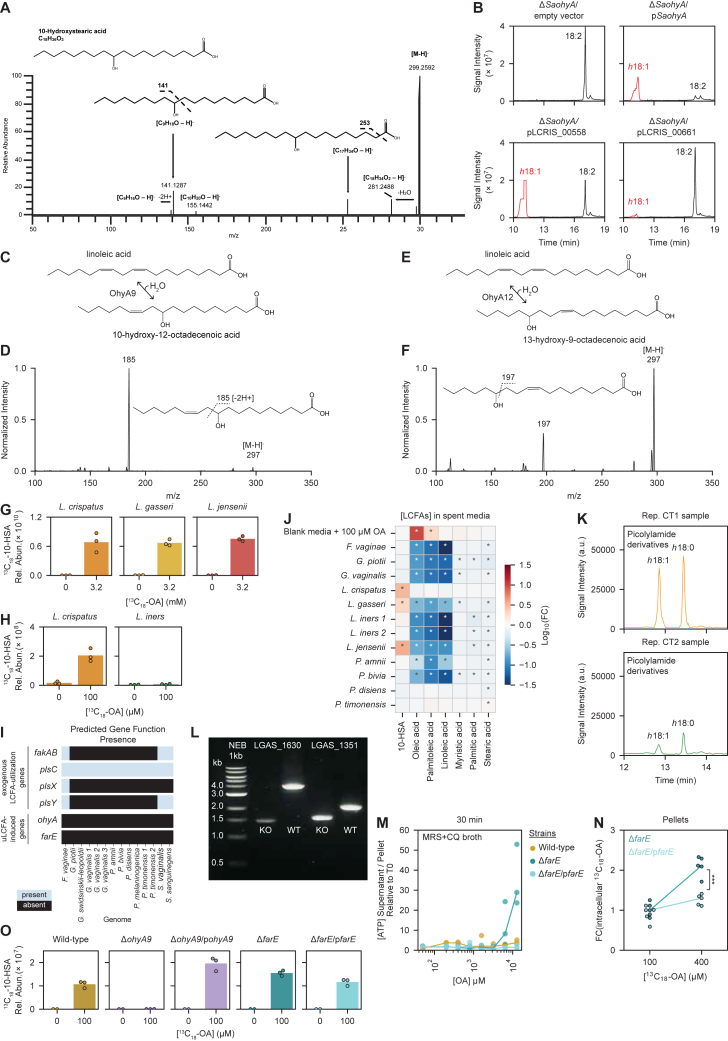
Detection of enzymatic products from *ohyA* orthologs and characterization of *ohyA9* and *farE* genetic knockouts in *L. gasseri*, related to Figures 4 and 5 (A) MS2 spectra with major fragmentation labels for 10-HSA standard (Ambeed, A125712-50MG). (B) Extracted ion chromatograms from supernatants of *ΔSaohyA*/empty vector, *ΔSaohyA*/p*SaohyA*, *ΔSaohyA*/pLCRIS_00558, and *ΔSaohyA*/pLCRIS_00661 cultured with LOA for 1 h. Annotated peaks include LOA (18:2) and the detected hydroxyFA (*h*18:1). (C) OhyA9 enzymatic activity reaction diagram with LOA substrate. (D) MS2 spectra with major fragmentation labels for the h18:1 peak from *ΔSaohyA*/pLCRIS_00661 cultured with LOA (lower right of Figure S4B), identified as 10-hydroxy-12-octadecenoic acid (*h*18:1). (E) OhyA12 enzymatic activity reaction diagram with LOA substrate. (F) MS2 spectra with major fragmentation labels for the h18:1 peak from *ΔSaohyA*/pLCRIS_00558 cultured with LOA (lower left of Figure S4B), identified as 13-hydroxy-9-octadecenoic acid. (G) ^13^C_18_-10-HSA concentrations in cell pellets of *L. crispatus*, *L. gasseri*, and *L. jensenii* cultured for 72 h in NYCIII broth with and without ^13^C_18_-OA (3.2 mM). The cell pellets are from the same cultures as the supernatants shown in Figure 3E. (H) ^13^C_18_-10-HSA concentrations in cell pellets of *L. crispatus* and *L. iners* cultured for 72 h in NYCIII broth with and without ^13^C_18_-OA (100 μM, which is a sublethal concentration for *L. iners*). The cell pellets are from the same cultures as the supernatants shown in Figure 3F. (I) Presence of gene functions predicted to encode oleate hydratase (*ohyA*) and putative fatty acid efflux pump (*farE*) activity in long-read sequenced, isolate genomes of the indicated FGT species. Presence of gene functions involved in exogenous fatty acid acquisition and utilization (*fakAB*, *plsC*, *plsX*, and *plsY*) is shown for comparison. (J) Untargeted lipidomics was performed on control (blank) media and spent media supernatants collected from strains of diverse FGT bacteria after 72 h of culture in NYCIII broth. Blank media supplemented with 100 μM OA is shown as a positive control. Changes in concentration of key LCFA metabolites for each bacterial species are shown as the log_10_(fold change) of their median relative abundances compared with control media. The plot depicts median fold change values for 5 technical replicates per condition. Statistical significance was determined by unpaired t test using the Bonferroni method to correct for multiple hypothesis testing (^∗^adjusted *p* < 0.05). (K) Representative *h*FA extracted ion chromatograms for human CVL samples with CT1 (*L. crispatus*-dominant, top) or CT2 (*L. iners*-dominant, bottom) bacterial communities, quantified via a targeted metabolomics approach employing picolylamine-based derivatization.^56^^,^^58^ (L) DNA gel of PCR products amplified from the primers flanking either LGAS_1630 (*farE*) or LGAS_1351 (*ohyA9*) from the *L. gasseri* ATCC 33323 wild-type (WT) strain and *ΔohyA9* and *ΔfarE* genetic knockout (KO) strains. Expected amplicon lengths were 3.9 kb for LGAS_1630 in WT, 2.0 kb for LGAS_1351 in WT, while in-frame gene deletions of LGAS_1351 in ΔohyA9 and of LGAS_1630 in ΔfarE each had expected amplicon lengths of 1.2 kb. KO strains were additionally WGS verified. (M) Cultures of *L. gasseri* WT, *L. gasseri* Δ*farE*, and *L. gasseri* Δ*farE*/p*farE* were grown to mid- to late-log phase and exposed to varying concentrations of OA, then ATP release assays were performed. (N) Detection of ^13^C-labeled OA (^13^C_18_-OA) in pellets of *L. gasseri* Δ*farE* and Δ*farE*/p*farE* genetic mutant strains treated with 100 and 400 μM ^13^C_18_-OA in NYCIII broth for 15 min. Pellets were washed two times with ice-cold PBS before sample preparation for targeted ^13^C_18_-OA detection. The fold change of [^13^C_18_-OA] was calculated by dividing the relative abundance of ^13^C_18_-OA detected in the 400 μM ^13^C_18_-OA condition for each technical replicate by the median relative abundance of ^13^C_18_-OA detected in 100 μM ^13^C_18_-OA condition. Significance of the difference in fold change was determined by unpaired t test (^∗∗∗^*p* < 0.001). Points represent 5 technical replicates per condition. (O) ^13^C_18_-10-HSA relative abundance in cell pellets from *L. gasseri* WT, *ΔohyA9*, *ΔohyA9*/p*ohyA9*, *ΔfarE*, and *ΔfarE*/p*farE* cultured for 24 h in NYCIII broth with and without ^13^C_18_-OA (100 μM). Pellets are from the same cultures as the supernatants in Figure 5D. Points represent 2 or 3 technical replicates per condition. (G and H) Points represent 3 technical replicates per condition.

To characterize enzymatic activities of predicted OhyA orthologs from FGT lactobacilli, we assessed function by heterologous expression in a well-characterized *S. aureus ohyA*-knockout strain (Δ*SaohyA*).^49^ We expressed the two most common *L. crispatus ohyA* orthologs (LCRIS_00661 and LCRIS_00558) in Δ*SaohyA*, along with the *S. aureus ohyA*^49^^*,*^^50^^*,*^^56^ (*SaohyA*) and empty vector as positive and negative controls. Complemented Δ*SaohyA* strains were cultured with OA or LOA, and then supernatants were assayed for OhyA products. Strains complemented with *SaohyA* or the OA-inducible *L. crispatus* OhyA ortholog (LCRIS_00661) hydrated *cis*-9 double bonds to generate the hydroxy FA (*h*FA) metabolite 10-hydroxystearic acid (10-HSA; *h*18:0) from OA and 10-hydroxy-12-octadecenoic acid (*h*18:1) from LOA (Figures 3B–3D and S4A–S4D). We therefore named this *L. crispatus* ortholog OhyA9. By contrast, the strain complemented with the *L. crispatus* OhyA ortholog (LCRIS_00558) hydrated the *cis*-12 double bond of LOA to produce 13-hydroxy-9-octadecenoic acid, so we named this ortholog OhyA12 (Figures 3C, S4B, S4E, and S4F). Thus, the phylogenetically distinct OhyA orthologs in *L. crispatus* possess related but distinct enzymatic activities.

To assess endogenous OhyA9 activity in FGT lactobacilli, we cultured representative strains in NYCIII broth with universally ^13^C-isotopically labeled OA (^13^C_18_-OA) and measured production of labeled 10-HSA (^13^C_18_-10-HSA) in supernatants and cell pellets. *L. crispatus*, *L. gasseri*, and *L. jensenii* each produced ^13^C_18_-10-HSA, confirming robust OhyA9 activity (Figures 3E and S4G). By contrast, *L. iners* failed to produce ^13^C_18_-10-HSA when cultured with sub-lethal ^13^C_18_-OA concentrations (Figures 3F and S4H). We also assessed common BV-associated (non-*Lactobacillus*) bacteria for OhyA activity. We generated and analyzed isolate genomes of *Fannyhessea* (formerly *Atopobium*) *vaginae*, *Sneathia vaginalis* (formerly *S. amnii*), *Sneathia sanguinegens*, and various *Gardnerella* and *Prevotella* species and grew several in mono-culture to assess for OhyA9 products. None of the genomes encoded predicted *ohyA* genes, and none of the cultured isolates produced 10-HSA (Figures S4I and S4J). These results confirmed non-*iners* FGT lactobacilli encode and express physiologically active OhyA9 enzymes, while *L. iners* and diverse BV-associated species do not.

### Women with microbiota dominated by non-*iners* lactobacilli have uniquely elevated vaginal concentrations of OhyA products

**Figure 4:**
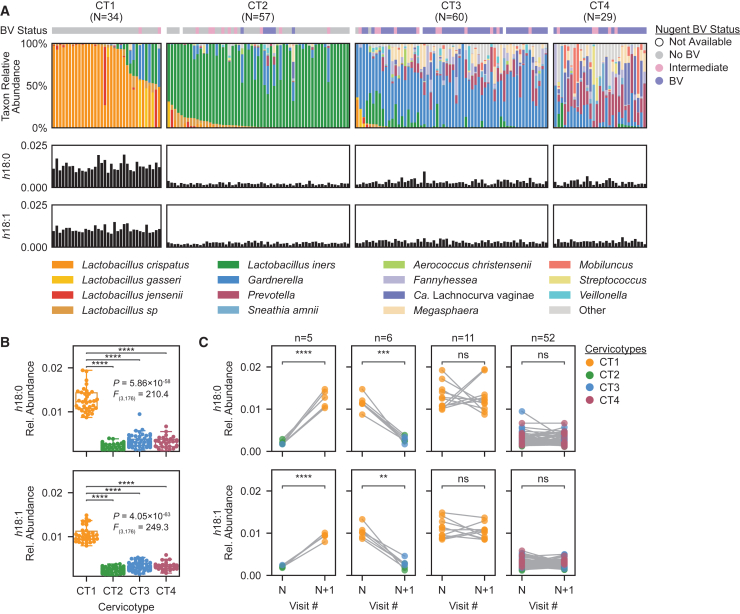
Women with non-*iners* lactobacilli have uniquely elevated vaginal concentrations of OhyA products (A) FGT microbiota composition of 180 distinct vaginal swab samples from 106 women, determined by bacterial 16S rRNA gene sequencing (top, stacked barplot) and classified into cervicotypes (CTs) as previously described.^10^^,^^36^ Middle and bottom bar plots show relative concentrations of h18:0 (10-HSA) and h18:1, respectively, in paired cervicovaginal lavage (CVL) samples. The top colorbar shows Nugent score-based BV status.^59^ (B) h18:0 (top) and h18:1 (bottom) concentrations within each CT for the samples in (A). Significance determined by one-way ANOVA with post hoc Tukey’s test; selected pairwise differences are shown (^∗∗∗∗^*p* < 0.0001; full statistical results in Table S3). Boxplots represent the 25^th^ and 75^th^ percentiles (lower and upper boundaries of boxes, respectively), the median (middle horizontal line), and measurements that fall within 1.5 times the IQR (whiskers). (C) Change in relative h18:0 (top) and h18:1 (bottom) concentrations within 74 paired serial samples in which microbiota transitioned to CT1 (*n* = 5), away from CT1 (*n* = 6), remained CT1 (*n* = 11), or remained non-CT1 (*n* = 52). Significance determined by paired t test on log-transformed values (^∗∗^*p* < 0.01; ^∗∗∗^*p* < 0.001; ^∗∗∗∗^*p* < 0.0001; ns: *p* ≥ 0.05). See also Figure S4.

We next assessed whether *Lactobacillus* OhyA enzymes were physiologically active *in vivo*. Humans lack OhyA,^53^ thus microbial OhyA activity was assessed by measuring concentrations of *h*FAs including *h*18:0 (10-HSA) and *h*18:1 in human cervicovaginal lavage (CVL) fluid. We tested 180 distinct samples from 106 participants in the Females Rising through Education, Support, and Health (FRESH) study, which enrolls South African women aged 18–23 years who provide cervicovaginal samples at 3-month intervals (Table S2).^57^ *h*FAs were quantified by targeted lipidomics using picolylamine-based derivatization^56^^,^^58^ (representative chromatograms in Figure S4K). Microbiota composition was profiled via bacterial 16S rRNA gene sequencing of matched vaginal swabs and was classified into distinct cervicotypes (CTs) using established criteria.^10^^,^^19^^,^^36^ Briefly, samples were classified as cervicotype 1 (CT1, *n* = 34 samples), defined by non-*iners Lactobacillus* species dominance; CT2 (*n* = 57), defined by *L. iners* dominance; CT3 (*n* = 60), defined by *Gardnerella* predominance; or CT4 (*n* = 29), consisting of diverse anaerobes, typically featuring high *Prevotella* abundance^10^ (Figure 4A). CT1 samples had significantly higher concentrations of *h*18:0 and *h*18:1 (4.8- and 3.6-fold higher median concentrations, respectively, compared with other CTs, Figure 4B). Remarkably, *h*18:0 and *h*18:1 concentrations provided near-perfect sensitivity and specificity in segregating CT1 from non-CT1 samples, with area-under-the-curve (AUC) values of 0.99 and 1.00, respectively, in receiver operating characteristic (ROC) analysis.

To further assess the relationship between microbiota composition and OhyA activity, we investigated how individual women’s *h*FA levels changed over time in 74 pairs of serially collected samples, including 5 pairs with transition from non-CT1 to CT1, 6 pairs with transition from CT1 to non-CT1, 11 pairs remaining in CT1, and 52 pairs remaining in non-CT1 communities. *h*18:0 and *h*18:1 concentrations significantly increased upon transition to CT1 (*p* = 0.00006, median 7.11-fold increase for *h*18:0; *p* = 0.00003, median 4.50-fold increase for *h*18:1) and significantly decreased upon transition away from CT1 (*p* = 0.000256, median 4.12-fold decrease for *h*18:0; *p* = 0.00118, median 3.88-fold decrease for *h*18:1; Figure 4C). By contrast, *h*FA levels did not significantly change in participants who remained in CT1 or in non-CT1 states (median fold changes ranging from 0.96 to 1.02 with *p >* 0.05 for each type of sample pair and *h*FA). Thus, OhyA product concentrations in cervicovaginal fluid tracked closely with non-*iners Lactobacillus* abundance over time, further confirming physiologic OhyA activity within the microbiota.

### *farE* is required for *Lactobacillus* resistance to OA inhibition, and *ohyA9* is required for 10-HSA production

**Figure 5:**
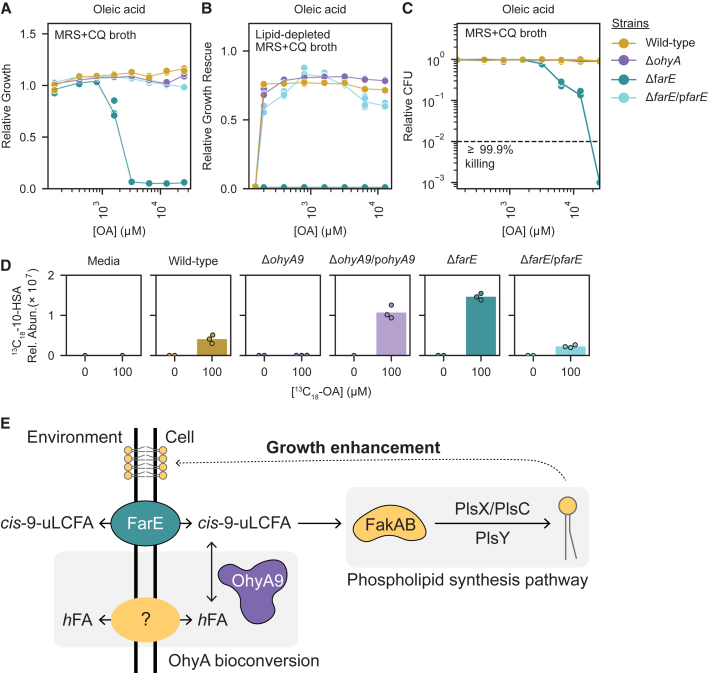
*farE* is required for resistance to OA inhibition, and *ohyA9* is required for 10-HSA production (A) Relative growth of *L. gasseri* ATCC 33323 wild-type (WT) and mutant strains, including knockouts of *ohyA9* (*ΔohyA9*) and *farE* (*ΔfarE*), and *ΔfarE* complemented with plasmid-overexpressed *farE* (*ΔfarE*/p*farE*), cultured in MRS + CQ broth supplemented with varying OA concentrations. (B) Relative growth rescue of *L. gasseri* WT and mutant strains in lipid-depleted MRS + CQ broth supplemented with varying OA concentrations. (C) MBC assay results for *L. gasseri* WT and mutant strains in MRS + CQ broth. (D) ^13^C_18_-10-HSA relative concentrations in blank media and supernatants from *L. gasseri* WT, *ΔohyA9*, *ΔohyA9* complemented with plasmid-overexpressed *ohyA9* (*ΔohyA9*/p*ohyA9*), *ΔfarE*, and *ΔfarE*/p*farE* cultured for 24 h in NYCIII broth with or without sub-inhibitory concentrations of ^13^C_18_-OA (100 μM). (E) Schematic depicting a proposed model for FarE and OhyA9 activity on exogenous *cis*-9-uLCFAs in non-*iners* FGT *Lactobacillus* species. (A–D) Points represent 2–3 technical replicates per condition. (A–C) Results are representative of ≥2 independent experiments. See also Figure S4.

We interrogated the relationship of *ohyA9* and *farE* to *Lactobacillus* OA growth phenotypes using a genetic approach. Tools to genetically modify *L. crispatus* and *L. iners* were not available, so we conducted these studies in *L. gasseri* since its *cis-*9-uLCFA responses shared close phenotypic, genomic, transcriptional, phylogenetic, and enzymatic similarity to *L. crispatus*. Adapting previously reported methods,^60^^,^^61^^,^^62^ we made genetic knockouts of *ohyA9* (*ΔohyA9*) and *farE* (*ΔfarE*) in the *L. gasseri* ATCC 33323 strain via in-frame, double homologous recombination-generated gene deletions (Figure S4L). We found that *farE*, but not *ohyA9*, was required for OA resistance (Figure 5A). *ΔfarE* also failed to grow in lipid-depleted MRS + CQ broth supplemented with OA, likely due to OA inhibition, while *ΔohyA9* showed similar growth enhancement as wild type (WT, Figure 5B). Complementing *ΔfarE* with plasmid-overexpressed *farE* (*ΔfarE*/p*farE*) fully restored OA resistance. However, OA inhibition of *L. gasseri ΔfarE* was less potently bactericidal than in *L. iners* (Figure 5C), and higher OA concentrations were required for membrane disruption in *ΔfarE* (Figure S4M), suggesting additional factors may contribute to *L. iners* susceptibility.

Since FarE is a putative fatty acid efflux pump, we hypothesized that *L. gasseri ΔfarE*’s OA susceptibility might reflect impaired regulation of intracellular OA concentrations. We therefore assessed ^13^C_18_-OA accumulation in *L. gasseri ΔfarE* and *ΔfarE*/p*farE* cultures exposed to 100 or 400 μM ^13^C_18_-OA. Raising exogenous ^13^C_18_-OA concentrations produced significantly greater fold increases in intracellular ^13^C_18_-OA in Δ*farE* than Δ*farE*/p*farE* (2.11- and 1.30-fold median increases, respectively; *p* = 0.00013), consistent with *farE*-dependent regulation of intracellular OA (Figure S4N).

To confirm *ohyA9*’s role in 10-HSA production, we grew *L. gasseri* WT, *ΔfarE*, *ΔfarE*/p*farE*, *ΔohyA9*, and *ΔohyA9* complemented with plasmid-overexpressed *ohyA9* (*ΔohyA9*/p*ohyA9*) in media with sub-inhibitory ^13^C_18_-OA concentrations, then measured ^13^C_18_-10-HSA in supernatants and cell pellets. As expected, ^13^C_18_-10-HSA production was fully ablated in *ΔohyA9* but restored in *ΔohyA9*/p*ohyA9* (Figures 5D and S4O). Interestingly, ^13^C_18_-10-HSA supernatant concentrations were higher than WT in *ΔfarE* but lower in *ΔfarE*/p*farE*, suggesting *farE* may influence 10-HSA production and/or export but is neither necessary nor sufficient for this process. We therefore establish two major response mechanisms in non-*iners* FGT *Lactobacillus* species: FarE prevents toxic intracellular *cis*-9-uLCFA accumulation, and OhyA9 bioconverts *cis*-9-uLCFAs to their 10-hydroxy-FA counterparts (Figure 5E).

### FGT *Lactobacillus* species are FA auxotrophs

**Figure 6:**
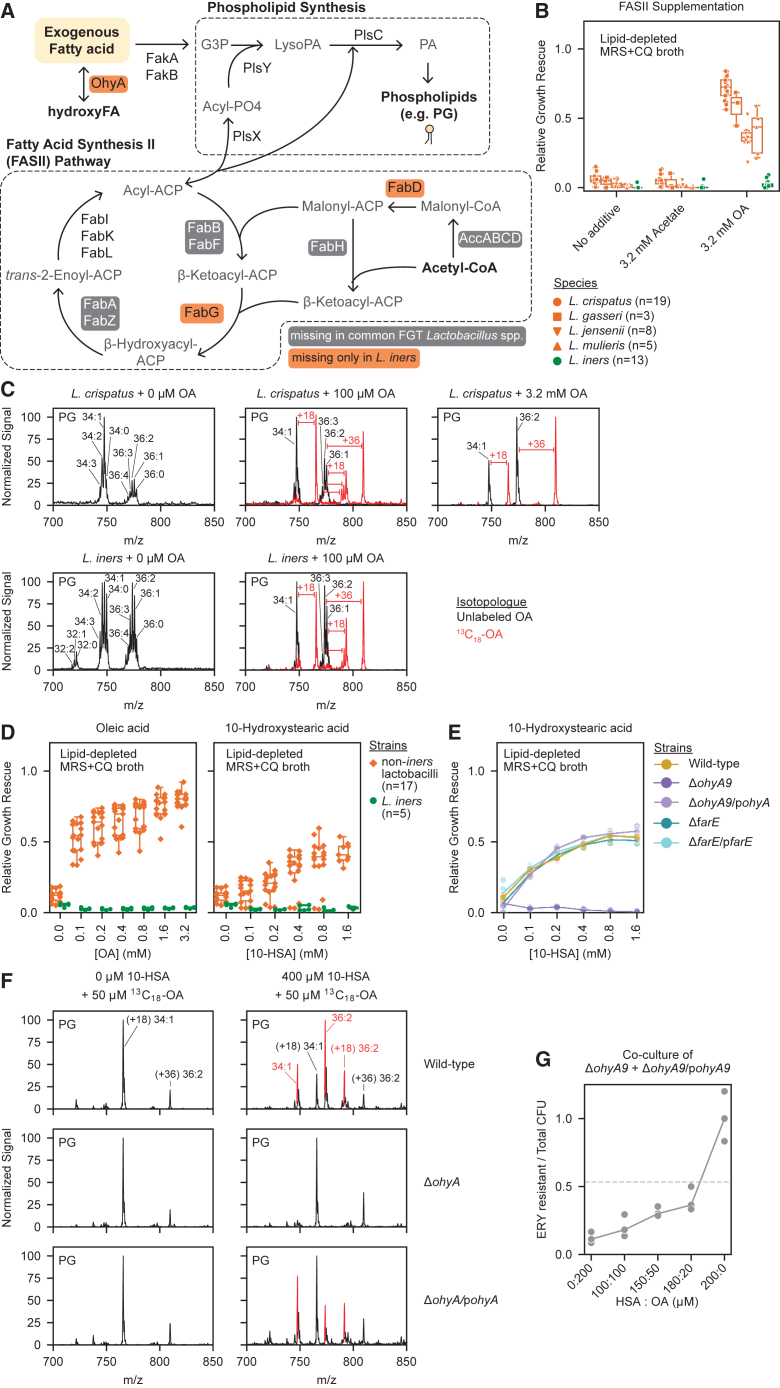
FGT lactobacilli are FA auxotrophs that exploit OA and its OhyA9-dependent derivative 10-HSA for phospholipid synthesis (A) Fatty acid synthesis II (FASII) and phospholipid synthesis pathways, annotated with predicted gene function presence in FGT *Lactobacillus* genomes. Predicted gene functions are annotated as missing in a species if absent in >50% of genomes. (B) Growth rescue of *L. crispatus* (*n* = 19), *L. gasseri* (*n* = 3), *L. iners* (*n* = 13), *L. jensenii* (*n* = 8), and *L. mulieris* (*n* = 5) strains in lipid-depleted MRS + CQ broth supplemented with acetate or OA (3.2 mM each), cultured for 72 h. (C) Phosphatidylglycerol (PG) profiles in cell pellets of *L. crispatus* (top) and *L. iners* (bottom) cultured for 72 h in NYCIII broth with no added OA (left) or supplemented with 100 μM (middle) or 3.2 mM (right, *L. crispatus* only) unlabeled OA (black) or ^13^C_18_-OA (red). Plots depict representative MS1 spectra. Predominant unlabeled isotopologs of major PG species (black) and the differences in mass/charge (m/z) ratio of their corresponding ^13^C-labeled isotopologs (red) are annotated. (D) Growth rescue of non-*iners* FGT *Lactobacillus* (*n* = 17) and *L. iners* (*n* = 5) strains in lipid-depleted MRS + CQ broth supplemented with varying concentrations of OA (left) or 10-HSA (right), cultured for 72 h. (E) Growth rescue of *L. gasseri* ATCC 33323 WT and mutant strains in lipid-depleted MRS + CQ broth supplemented with varying concentrations of 10-HSA, cultured for 24 h. (F) Detection of PG lipids in pellets from *L. gasseri* WT (top), *ΔohyA9* (middle), and *ΔohyA9*/p*ohyA9* (bottom), cultured for 24 h in lipid-depleted MRS + CQ broth containing 50 μM ^13^C_18_-OA with (right) or without (left) 400 μM unlabeled 10-HSA (growth shown in Figure S5G). Plots depict representative MS1 spectra with major ^13^C-labeled (black) and partially labeled or unlabeled (red) PG species annotated. (G) *L. gasseri* genetic mutants strains, Δ*ohyA9* and Δ*ohyA9*/p*ohyA9*, were co-cultured in lipid-depleted MRS + CQ broth supplemented with varying concentrations of OA and 10-HSA, but no erythromycin selection. After 18 h, the ratio of CFU on MRS agar plates with and without erythromycin (respectively representing the Δ*ohyA9*/p*ohyA9* CFU relative to the total CFU) and relative growth (Figure S5I) were determined. The dotted line represents the input CFU ratio. (B, D, and E) Relative growth rescue was calculated as growth relative to median OD600 measurement in non-lipid-depleted MRS + CQ broth. (B and D) Points represent median relative growth for 3 technical replicates per condition. Boxplots represent the 25^th^ and 75^th^ percentiles (lower and upper boundaries of boxes, respectively), the median (middle horizontal line), and measurements that fall within 1.5 times the IQR (whiskers). (E) Points represent 3 technical replicates per condition and are representative of ≥2 independent experiments. See also Figure S5.

**Figure S5:**
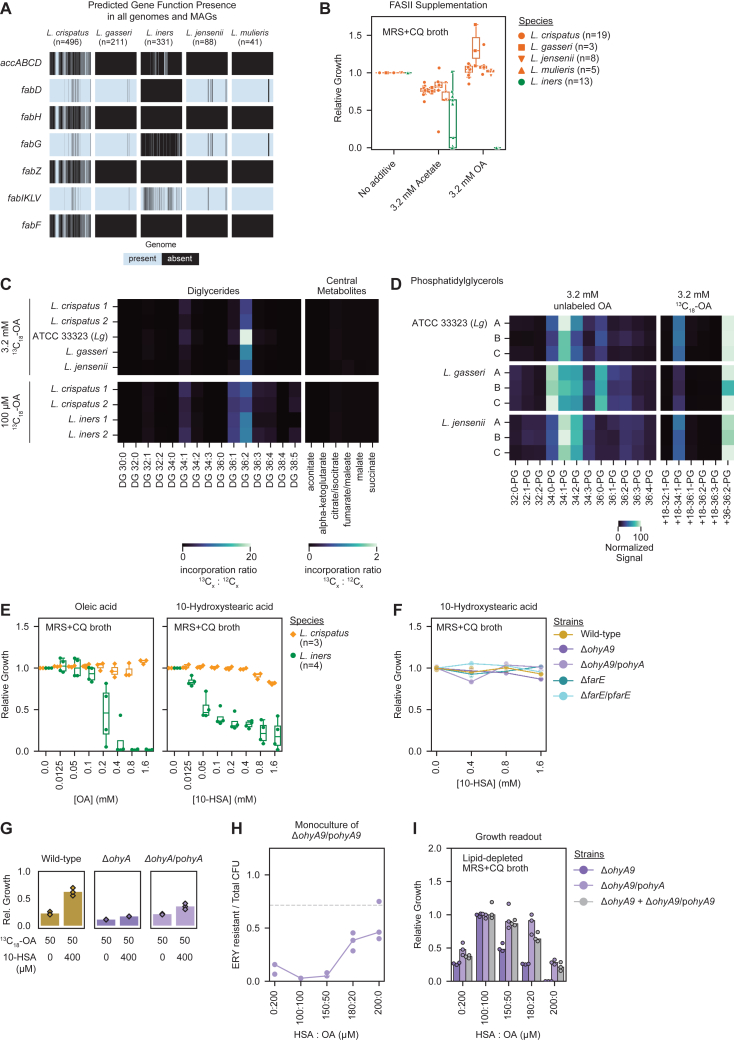
Genomic analysis of FASII pathway in FGT *Lactobacillus* genomes, OA isotope tracing in cultured FGT lactobacilli, and 10-HSA growth effects, related to Figure 6 (A) Presence of gene functions predicted to encode FASII pathway genes in isolate genomes and MAGs of the indicated FGT *Lactobacillus* species (*n* = 1,167). (B) Relative growth of diverse *L. crispatus* (*n* = 19), *L. gasseri* (*n* = 3), *L. iners* (*n* = 13), *L. jensenii* (*n* = 8), and *L. mulieris* (*n* = 5) strains in MRS + CQ broth supplemented with 3.2 mM acetate or 3.2 mM OA. Growth was measured by OD600 after 72 h of culture. (C) Heatmap representing the median incorporation ratio of ^13^C_18_-OA in detected diglycerides and central metabolites involved in the tricarboxylic acid (TCA) cycle in cell pellets from representative strains of FGT *Lactobacillus* species. Bacteria were cultured for 72 h in NYCIII broth with 3.2 mM (top) or 100 μM (bottom). ^13^C_18_-OA incorporation ratio was calculated as the signal from the detected ^13^C-labeled metabolite relative to the signal of the detected unlabeled metabolite. (D) Heatmap representing the normalized signal of detected unlabeled and labeled phosphatidylglycerol in cell pellets from representative strains of FGT *Lactobacillus* species. Bacteria were cultured for 72 h in NYCIII broth with 3.2 mM unlabeled OA (left) or ^13^C_18_-OA (right). Each row labeled A, B, and C represents a replicate culture of the indicated condition. (E) Relative growth of *L. gasseri* WT and mutant strains in MRS + CQ broth supplemented with varying concentrations of 10-HSA. Growth was measured by OD600 after 24 h of culture. (F) Relative growth of *L. crispatus* (*n* = 3) and *L. iners* (*n* = 4) strains in MRS + CQ broth supplemented with varying concentrations of OA (left) or 10-HSA (right). Growth was measured by OD600 after 72 h of culture. (G) *L. gasseri* genetic mutant strains (WT, *ΔohyA9*, and *ΔohyA9*/p*ohyA9*) were grown for 24 h in lipid-depleted MRS + CQ broth supplemented with ^13^C_18_-OA concentration (50 μM) alone and in combination with unlabeled 10-HSA (400 μM, corresponding to the isotopic tracing data in Figure 6F). Relative growth was calculated relative to the median OD600 measurement in non-lipid-depleted MRS + CQ broth. (H) *L. gasseri* Δ*ohyA9*/p*ohyA9* was grown in lipid-depleted MRS + CQ broth supplemented with varying concentrations of OA and 10-HSA, but without erythromycin selection (the p*ohyA9* plasmid encodes erythromycin resistance). After 18 h of culture, the ratio of CFU on MRS agar plates with and without erythromycin (respectively representing the amount of the viable Δ*ohyA9*/p*ohyA9* strain relative to total bacteria) was determined. (I) Growth (determined by OD600) of *L. gasseri* genetic mutants strains Δ*ohyA9* and Δ*ohyA9*/p*ohyA9* grown in mono-culture or mixed and co-cultured in lipid-depleted MRS + CQ broth supplemented with varying concentrations of OA and 10-HSA, without erythromycin selection. Relative growth was calculated relative to the median OD600 measurement in the 100 μM HSA: 100 μM OA condition. The cultures correspond to the experiment shown in Figure 6G. (B, E, and F) Relative growth was calculated relative to the median OD600 measurement in the no supplementation control. (B and E) Boxplots represent the 25^th^ and 75^th^ percentiles (lower and upper boundaries of boxes, respectively), the median (middle horizontal line), and measurements that fall within 1.5 times the IQR (whiskers). (B and F) Points represent the median of 3 technical replicates per condition. (E) Points represent 3 technical replicates per condition.

We next investigated *Lactobacillus* FA synthesis to identify potential mechanisms of OA-dependent growth enhancement. FGT *Lactobacillus* genomes were largely predicted to lack an intact fatty acid synthesis II (FASII) pathway, including genes predicted to encode AccABCD, FabH, FabB/F, and FabA/Z (Figures 6A and S5A).^63^ *L. iners* also lacked predicted genes for FabG and FabD. A minority of *L. crispatus* genomes contained a predicted intact FASII pathway, but—in contrast to OA—supplementing lipid-depleted media with acetate (a precursor to the FASII initiating molecule, acetyl-coenzyme A) failed to rescue growth of all experimental FGT *Lactobacillus* strains, including *L. crispatus* strains with predicted FASII pathways. Adding acetate to lipid-replete media did not substantially inhibit non-*iners Lactobacillus* species, demonstrating that acetate’s failure to rescue lipid-dependent growth was not due to direct toxicity (*n* = 45 strains; Figures 6B and S5B). Collectively, these results show FGT *Lactobacillus* species are FA auxotrophs.

We next traced the fate of ^13^C_18_-OA in cultured bacteria. Genomic analysis predicted all major FGT *Lactobacillus* species possessed genes required to utilize exogenous LCFAs for phospholipid synthesis (*fakAB* and *plsCXY*, Figure 2C). Isotopic tracing confirmed OA incorporation into structural lipid metabolites including phosphatidylglycerol (PG) and diglyceride lipids in *L. crispatus*, *L. iners*, *L. gasseri*, and *L. jensenii* (Figures 6C and S5C). Incorporation into PGs was primarily observed in PG 34:1, PG 36:1, and PG 36:2, which became the predominant PGs when *L. crispatus*, *L. gasseri*, and *L. jensenii* were grown in high (*L. iners*-inhibitory) OA concentrations, demonstrating these organisms’ immense versatility to utilize exogenous OA for membrane synthesis (Figures 6C and S5D). By contrast, these lactobacilli lacked predicted beta-oxidation pathways to exploit LCFAs for central energy metabolism, and we observed no ^13^C label incorporation from OA in central energy metabolites (Figure S5C). Thus, major FGT *Lactobacillus* species—including *L. iners*—require exogenous LCFAs to build membranes, but only *farE*-harboring (non-*iners*) species can resist and utilize high concentrations of free exogenous OA.

### Non-*iners* FGT lactobacilli can exploit 10-HSA for growth via *ohyA9*-dependent conversion to OA

Since concentrations of 10-HSA (*h*18:0) and related *h*FAs were uniquely elevated in CT1 (Figure 4B) and OhyA enzymes can act bidirectionally,^64^^,^^65^ we hypothesized that OhyA9 enables non-*iners* FGT lactobacilli to convert 10-HSA to OA for phospholipid synthesis, providing a unique uLCFA source inaccessible to *ohyA*-deficient bacteria. We therefore assessed the effects of supplementing lipid-depleted media with 10-HSA instead of OA. As hypothesized, non-*iners* FGT *Lactobacillus* species (*n* = 17 strains) exhibited 10-HSA-dependent growth enhancement, whereas 10-HSA inhibited *L. iners* growth (*n* = 5 strains, Figure 6D), albeit with lower potency than OA (∼4-fold higher MIC; Figure S5E). To confirm that 10-HSA-driven growth enhancement required OhyA9, we grew *L. gasseri* WT, *ΔfarE*, *ΔfarE*/p*farE*, *ΔohyA9*, and *ΔohyA9*/p*ohyA9* strains in 10-HSA-supplemented, lipid-depleted media. 10-HSA failed to support *ΔohyA9* growth, but growth was rescued in *ΔohyA9*/p*ohyA9* (Figure 6E), confirming *ohyA9*-dependence. 10-HSA did not inhibit *L. gasseri ΔohyA9* in non-lipid-depleted media, indicating that its failure to grow in lipid-depleted conditions was not due to direct toxicity (Figure S5F).

Isotopically labeled 10-HSA was not commercially available to directly test whether OhyA9 could convert 10-HSA to OA for phospholipid synthesis. We therefore investigated this hypothesis by growing *L. gasseri* WT, *ΔohyA9*, and *ΔohyA9*/p*ohyA9* in lipid-depleted media supplemented with ^13^C_18_-OA alone or with unlabeled 10-HSA (Figure S5G), then measuring incorporation of labeled versus unlabeled OA into major PG molecules, including PG 34:1 and PG 36:2 (Figure 6F). All strains almost exclusively produced PG 34:1 and PG 36:2 containing ^13^C_18_-OA when cultured in ^13^C_18_-OA alone. However, adding unlabeled 10-HSA led WT and *ΔohyA9*/p*ohyA9* (but not *ΔohyA9*) strains to produce PGs containing unlabeled OA as well. Thus, *ohyA9*-harboring lactobacilli can exploit 10-HSA for growth by converting it to OA for phospholipid synthesis.

We hypothesized that *ohyA9* would offer a fitness advantage in high 10-HSA environments. Indeed, the *ΔohyA9*/p*ohyA9* strain retained the p*ohyA9* plasmid even without antibiotic selection when cultured in lipid-depleted media supplemented with 10-HSA alone or a high ratio of 10-HSA to OA (Figure S5H). To further assess *ohyA*’s fitness benefits in high 10-HSA settings, we co-cultured *ΔohyA9* and *ΔohyA9*/p*ohyA9* in lipid-depleted media lacking antibiotic selection but supplemented with 10-HSA and/or OA in varying ratios, then measured strain competitive index by selective plating. *ΔohyA9* robustly outcompeted *ΔohyA9*/p*ohyA9* in OA alone, likely due to the metabolic burden from p*ohyA9*’s strong constitutive promoter. However, *ΔohyA9*/p*ohyA9* displayed increasing competitive fitness with increasing ratios of 10-HSA to OA, and *ΔohyA9*/p*ohyA9* fully outcompeted *ΔohyA9* in media supplemented with 10-HSA alone (Figures 6G and S5I). Thus, *ohyA9* enables non-*iners* FGT lactobacilli to establish high levels of 10-HSA and related *h*FAs in the vaginal environment and also enables them to exclusively exploit these *h*FAs for phospholipid synthesis, a competitive strategy we refer to as biochemical nutrient sequestration.

### OA inhibits key BV-associated bacteria, including MTZ-resistant strains

**Figure 7:**
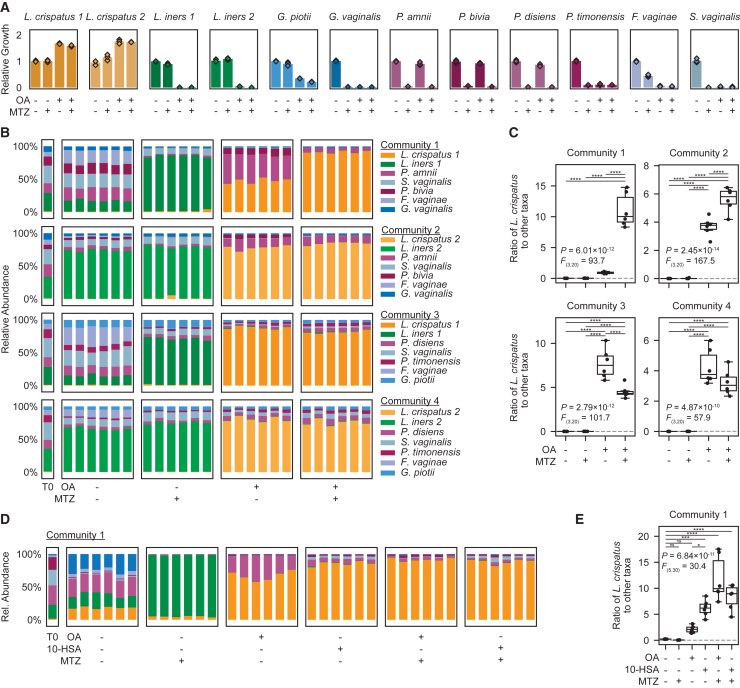
OA and 10-HSA treatments (with or without MTZ) shift *in vitro* BV-like communities toward *L. crispatus* dominance (A) Relative growth of the indicated species in NYCIII broth with or without metronidazole (MTZ; 50 μg/mL) and/or OA (3.2 mM), cultured for 72 h. Points represent 3 technical replicates per condition. (B) Relative bacterial abundance in defined BV-like communities grown for 72 h in NYCIII broth with or without MTZ (50 μg/mL) and/or OA (3.2 mM). (C) Ratios of *L. crispatus* to the sum of all other taxa in the communities in (B). (D) Relative bacterial abundance in a defined BV-like community grown for 72 h in NYCIII broth with OA (3.2 mM) or 10-HSA (1.6 mM), with or without metronidazole (MTZ; 50 μg/mL). (E) Ratios of *L. crispatus* to the sum of all other taxa in the communities in (D). (B–E) Plots depict 6 technical replicates per condition. (B and D) Compositions of the cultured communities and input mixture (T0) were determined by 16S rRNA gene sequencing. (C and E) Dotted lines represent the ratios in the input mixtures (T0). Boxplots represent the 25^th^ and 75^th^ percentiles (lower and upper boundaries of boxes, respectively), the median (middle horizontal line), and measurements that fall within 1.5 times the IQR (whiskers). Between-group differences were determined by one-way ANOVA with post hoc Tukey’s test; selected significant pairwise differences are shown (ns: not significant; ^∗^*p* ≤ 0.05; ^∗∗∗^*p* < 0.001; ^∗∗∗∗^*p* < 0.0001; full statistical results in Table S3). See also Figures S6 and S7.

**Figure S6:**
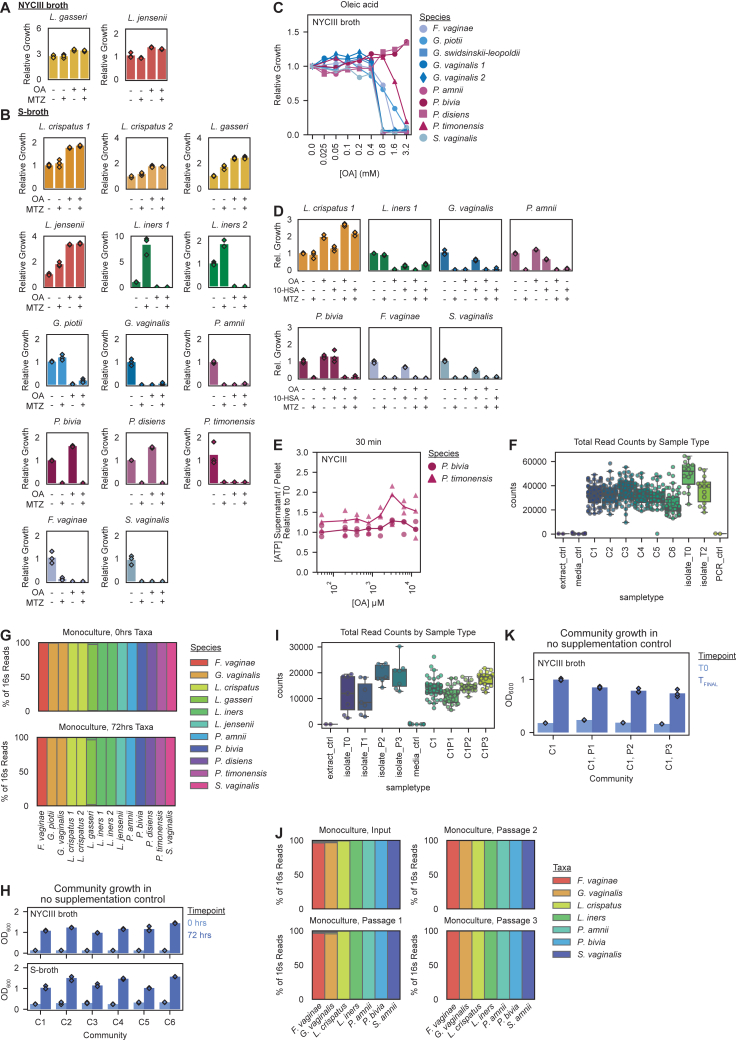
Effects of OA and MTZ on growth of FGT bacterial species and defined, *in vitro* BV-like bacterial community experiment controls, related to Figure 7 (A) Relative growth of representative *L. gasseri* and *L. jensenii* strains in NYCIII broth with or without MTZ (50 μg/mL) and/or OA (3.2 mM). (B) Relative growth of the indicated bacterial species in S-broth with or without MTZ (50 μg/mL) and/or OA (3.2 mM). (C) Relative growth of representative *F. vaginae, G. piotii, G. swindsinskii-leopoldii, G. vaginalis* (2 strains), *P. amnii*, *P. bivia*, *P. timonensis*, and *S. vaginalis* strains in NYCIII broth with varying concentrations of OA. Points represent median relative growth for 3 technical replicates per condition. (D) Relative growth of the indicated bacterial species in NYCIII broth with OA (3.2 mM) or 10-HSA (1.6 mM), each alone or in combination with metronidazole (MTZ; 50 μg/mL), after 72 h of culture. (E) Cultures of *P. bivia* and *P. timonensis* were grown to mid- to late-log phase and then exposed to varying concentrations of OA, then ATP release assays were performed. (F) Total sequencing read counts from mock BV-like community experiments per sample type, including extraction controls (extract_ctrl), blank media controls (media_ctrl), community samples (C1–C6 corresponding to samples from Figures 7B, 7C, S7A, and S7B), input bacterial isolate mono-cultures (isolate_T0), 72-h cultured bacterial isolate mono-cultures (isolate_T2), and no template PCR controls (PCR_ctrl). (G) Taxonomic composition of bacterial isolate mono-cultures used to construct defined communities in Figures 7B, 7C, S7A, and S7B, determined by 16S rRNA gene sequencing to confirm mono-culture purity. Plot shows data for input (top) isolates and for the corresponding 72-h mono-culture controls (bottom). (H) OD600 for each total community (C1–C6) at 0 and 72 h of culture in NYCIII broth (top) and S-broth (bottom), corresponding to community cultures in Figures 7B, 7C, S7A, and S7B. (I) Total sequencing read counts from mock BV-like community experiments per sample type, including extraction controls (extract_ctrl), blank media controls (media_ctrl), community samples (C1 corresponding to samples from Figures 7D and 7E; C1P1–3 corresponding to samples from Figure S7C), input bacterial isolate mono-cultures (isolate_T0), and cultured bacterial isolate mono-cultures (isolate_T1, isolate_P2, and isolate_P3). (J) Taxonomic composition of bacterial isolate mono-cultures used to construct the defined communities in Figures 7D, 7E, and S7C, determined by 16S rRNA gene sequencing to confirm mono-culture purity. Plot shows data for input (top) isolates and the corresponding passaged cultures (passage 1–3), each cultured for 48 h (bottom). (K) OD600 for each total community (C1 corresponding to Figures 7D and 7E; C1P1–3 corresponding to Figure S7C) at 0 h for all communities, 72 h for C1, and 48 h for C1P1–3 of culture in NYCIII broth. (A–D) Growth was measured by OD600 after 72 h of culture. Relative growth was calculated relative to median OD600 measurement in the no-additive control. (A–E) Points represent 3 technical replicates per condition. (F and I) Boxplots represent the 25^th^ and 75^th^ percentiles (lower and upper boundaries of boxes, respectively), the median (middle horizontal line), and measurements that fall within 1.5 times the IQR (whiskers).

To further investigate OA’s effects within the FGT microbiota, we characterized its impact on various non-*Lactobacillus* FGT bacteria. Analysis of isolate genomes from diverse, BV-associated species identified no putative *farE* orthologs (Figure S4I). Mono-cultures of *Gardnerella vaginalis*, *Gardnerella piotii*, *Fannyhessea* (formerly *Atopobium*) *vaginae*, *Sneathia vaginalis* (formerly *amnii*), and *Prevotella timonensis* were all robustly inhibited by OA, while other *Prevotella* species, including *P. bivia*, *P. amnii*, and *P. disiens*, were largely unaffected in NYCIII broth (Figures 7A and S6A). Results in S-broth were similar, except that OA inhibited *P. amnii* (Figure S6B). Interestingly, OA inhibited MTZ-resistant strains of *G. piotii* and *F. vaginae*, suggesting potential utility in targeting MTZ-resistant, BV-associated species. Median OA MICs for *Gardnerella* were approximately 2-fold greater than for *L. iners* (Figure S6C). By contrast, 10-HSA had little or no inhibitory effect against these species (Figure S6D). To assess whether OA inhibited BV-associated bacteria (some of which are gram-negative) via membrane disruption, we performed ATP release assays for *P. timonensis* and *P. bivia*, which were OA-susceptible and resistant, respectively. Neither species showed consistent increases in ATP release upon OA treatment, suggesting OA inhibits gram-negative anaerobes via a different mechanism than gram-positive lactobacilli (Figure S6E).

### OA and 10-HSA shift *in vitro* BV-like bacterial communities toward *L. crispatus* dominance, alone or with MTZ

**Figure S7:**
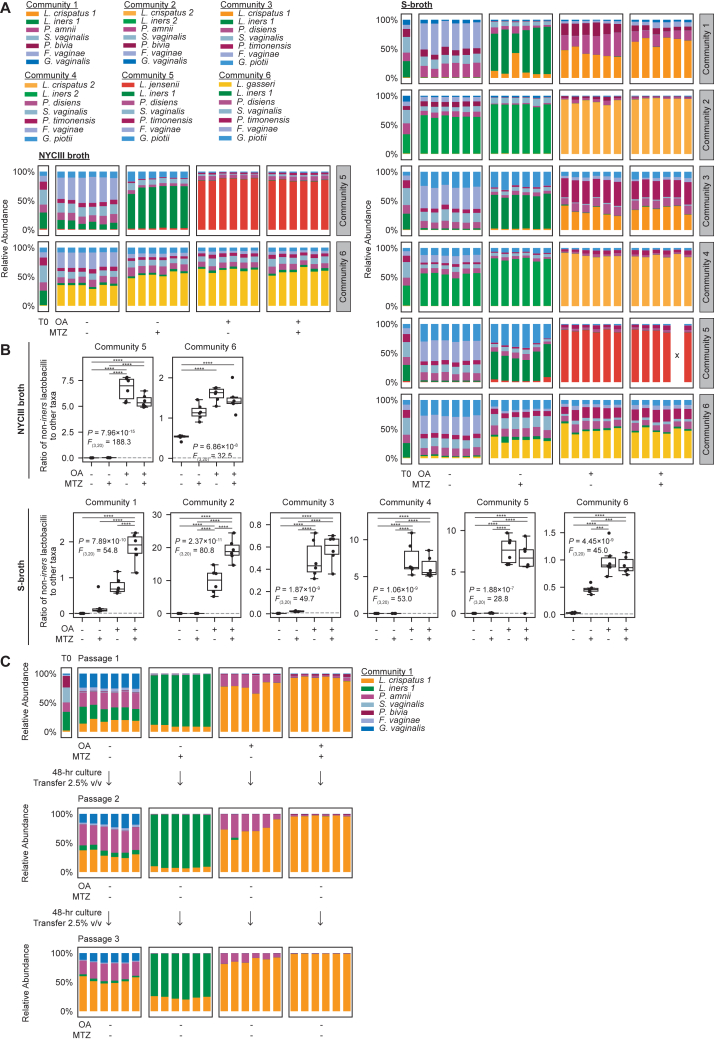
Community compositions for experiments in S-broth and OA-shifted *L. crispatus* dominance is stable over multiple passages *in vitro*, related to Figure 7 (A) Relative bacterial abundance in defined BV-like communities grown for 72 h in NYCIII broth or S-broth with or without MTZ (50 μg/mL) and/or OA (3.2 mM). Composition of the cultured communities and of the input mixtures (T0) was determined by bacterial 16S rRNA gene sequencing (see also Figures 7B and 7C). Plots depict 6 technical replicates per condition. Sequencing reads were not recovered from a single technical replicate of community 5 cultured in S-broth with MTZ and OA (due to a failed PCR reaction). This replicate is marked with an “x” in the plot, and its corresponding low read count is shown in Figure S6F. (B) Ratios of non-*iners* FGT *Lactobacillus* species taxa to the sum of all other taxa in the mock communities shown in (A). The gray dotted lines represent the ratios measured in the input inocula (T0). Boxplots represent the 25^th^ and 75^th^ percentiles (lower and upper boundaries of boxes, respectively), the median (middle horizonal line), and measurements that fall within 1.5 times the IQR (whiskers). Between-group differences were determined by one-way ANOVA with post hoc Tukey’s test; selected significant pairwise differences are shown (^∗∗∗^*p* < 0.001, ^∗∗∗∗^*p* < 0.0001; full statistical results in Table S3). (C) Relative bacterial abundance in a defined BV-like community grown for 48 h in NYCIII broth with or without OA (3.2 mM) alone or in combination with MTZ (50 μg/mL; “P1”). After 48 h of culture, 2.5% v/v of each technical replicate was passaged into NYCIII broth without additives (“P2”) and cultured for another 48 h. Each replicate culture was then passaged once more into NYCIII broth without additives (“P3”). Composition of the cultured communities and of the input mixture (T0) was determined by bacterial 16S rRNA gene sequencing. Plots depict 6 technical replicates per condition.

OA’s effects on FGT bacteria suggested therapeutic potential for shifting vaginal bacterial communities toward *L. crispatus* dominance, alone or in combination with MTZ. We tested this hypothesis using an established approach employing defined, BV-like bacterial communities *in vitro*.^36^ The input communities, comprising predominantly BV-associated bacteria with low abundance of *L. iners* and *L. crispatus* in ratios typical of BV, were cultured in NYCIII broth with or without OA and/or MTZ (Figures 7B and 7C). Community composition was assessed by 16S rRNA gene sequencing, and growth was confirmed by optical density at 600 nm (OD600) (Figures S6F–S6H). Untreated communities maintained BV-like compositions, while MTZ-treated communities became dominated by *L. iners*, mimicking MTZ’s effects in human BV^21^^,^^25^^,^^26^^,^^27^^,^^28^ (Figures 7B and 7C). By contrast, OA significantly promoted *L. crispatus* while suppressing *L. iners* and most BV-associated species, but failed to suppress some *Prevotella*. Combining OA with MTZ preserved or enhanced *L. crispatus* dominance compared with OA alone, while reducing *Prevotella*. Results were similar for communities containing *L. gasseri* or *L. jensenii* instead of *L. crispatus*, and culturing the same communities in S-broth produced similar results (Figures S7A and S7B). To assess durability of OA effects in this model, we serially passaged treated communities into fresh broth media lacking OA and MTZ. OA-dependent enrichment of *L. crispatus* remained durable even after multiple passages into media without supplemented OA (Figures S6I–S6K and S7C). Adding 10-HSA instead of OA to a mock BV-like community also promoted *L. crispatus* dominance (Figures 7D, 7E, and S6I–S6K). Thus, OA and 10-HSA effects in this model demonstrate potential to improve BV therapy.

## Discussion

This study describes differences in fatty acid metabolism among FGT *Lactobacillus* species that reveal fundamental principles of nutrient utilization in the microbiota and suggest metabolite-based treatment strategies for BV. Specifically, we found that *cis-*9-uLCFAs—exemplified by OA—selectively inhibit *L. iners* and key BV-associated bacteria while robustly promoting growth of *L. crispatus* and other health-associated, non-*iners* FGT lactobacilli. Genomic and transcriptomic analyses identified a core set of OA-induced *Lactobacillus* genes that *L. iners* lacks, including an oleate hydratase (*ohyA*) and a putative fatty acid efflux pump (*farE*). We experimentally confirmed enzymatic activity of *Lactobacillus* OhyA orthologs and showed that OhyA products, including 10-HSA, were uniquely elevated in cervicovaginal fluid of women with *L. crispatus*-dominated FGT microbiota. Genetically manipulating FGT lactobacilli has historically been a key challenge, but we succeeded in making and complementing knockouts of *farE* and *ohyA9*. Characterizing the knockouts revealed that *farE* was required for OA resistance, while *ohyA9* was required for reversible interconversion of OA and 10-HSA. We further showed that FGT lactobacilli (including *L. iners*) were FA auxotrophs requiring exogenous LCFAs such as OA for phospholipid synthesis, but only *farE*-harboring (i.e., non-*iners*) species could survive and utilize high concentrations of free OA. Non-*iners* lactobacilli could also exploit 10-HSA for phospholipid production via *ohyA9*-dependent conversion to OA, providing a competitive advantage by biochemically sequestering OA in a derivative form inaccessible to non-*ohyA*-harboring bacteria. Finally, treatment with OA or 10-HSA robustly shifted *in vitro* BV-like bacterial communities toward *L. crispatus* dominance. Together, these results advance mechanistic understanding of FGT *Lactobacillus* metabolism and competition within the microbiota while identifying therapeutic strategies for BV.

Our description of uLCFA-mediated inhibition of *L. iners* adds to existing knowledge about uLCFA antimicrobial properties in mammalian-adapted bacteria.^41^ Antimicrobial activity of host-produced free FAs contributes to innate defense in human skin, oral mucosa, and likely other mucosae.^43^^,^^66^^,^^67^ uLCFA antibacterial potency and mechanisms of action vary between bacterial strains and species. For example, POA and sapienic acid potently inhibit *S. aureus* via cell membrane disruption, causing leakage of low-molecular-weight solutes and interfering with energy metabolism.^42^ Similarly, sapienic and lauric acid disrupt the cell membrane and lyse *Porphyromonas gingivalis*.^68^ By contrast, high OA and LOA concentrations are reported to bacteriostatically inhibit certain *S. aureus* strains by inhibiting the FASII biosynthetic enzyme FabI.^69^ We find that OA induces rapid membrane integrity loss and lysis in *L. iners*, consistent with a membrane disruption mechanism.

Our finding of a conserved *cis*-9-uLCFA response involving OhyA, FarE, and TetR in non-*iners* FGT lactobacilli aligns with reported uLCFA resistance mechanisms in other organisms. For example, *S. aureus* FarE reportedly confers resistance to LOA and arachidonic acid via uLCFA efflux,^70^ and Tet38 (a separate *S. aureus* fatty acid efflux pump) promotes POA resistance.^71^ TetR mutations that increased FarE expression in *S. aureus* enhance LOA resistance,^70^ while TetR mutations in *Lactobacillus johnsonii* have been linked to evolved LOA resistance (although these mutations’ effects on TetR function were not directly assessed).^72^ A functionally similar multiple transferable resistance CDE (MtrCDE) efflux system in *Neisseria gonorrhoeae* exports hydrophobic molecules such as LCFAs and enhances survival in FA-rich environments, including the murine FGT.^73^ Thus, our findings add to a body of literature showing FA efflux pumps offer an important LCFA resistance mechanism and fitness advantage in LCFA-rich environments. We speculate that *L. iners*’s lack of *farE* and associated inability to prevent toxic uLCFA accumulation contributes to its susceptibility. By contrast, *ohyA9* was not required for *L. gasseri cis*-9-uLCFA resistance in our experimental conditions, contrasting with findings in *S. aureus* and *S. pyogenes*, where *ohyA* provided POA resistance.^49^^,^^52^ Thus, *ohyA*’s role in resistance to uLCFAs may be species- or context-dependent.

In addition to their ability to resist uLCFA toxicity, we find that non-*iners* FGT *Lactobacillus* species are FA auxotrophs that derive growth benefits from exogenous OA and (in *ohyA9*-dependent fashion) its derivative 10-HSA. These species thus depend on LCFAs obtained from their environment, host, or fellow microbes. We show that several major non-*Lactobacillus* FGT bacteria fail to produce or actively deplete exogenous LCFAs. Since the FGT microbiota is not in direct contact with dietary FAs except via host absorption and metabolism, we propose that host-derived FAs are likely the primary FA source for FGT lactobacilli.

Our findings highlight important nutrient requirements and metabolic niche characteristics of human FGT lactobacilli but also hold implications for other microbiota contexts. Like FGT *Lactobacillus* species, other important mammalian-associated *Lactobacillus* species, including *L. johnsonii*, *L. helveticus*, and *L. acidophilus*, are FA auxotrophs.^74^^,^^75^ Our genomic analysis reveals that all vertebrate-associated *Lactobacillus* species^55^ except *L. iners* possess both *ohyA* and *farE*. We show that *ohyA*-harboring FGT lactobacilli can modify *cis*-9-uLCFAs to generate high concentrations of *h*FAs like 10-HSA in their environment and can convert 10-HSA back into OA for membrane phospholipid synthesis. This reversible *ohyA*-dependent bioconversion—which we term biochemical nutrient sequestration—provides a fitness advantage in competition with non-*ohyA*-expressing bacteria, which are unable to exploit *h*FAs. Biochemical sequestration of uLCFAs adds to previously described microbial mechanisms for molecularly encrypting nutrients to prevent access by competing species.^76^ For example, bacteria use siderophores to bind and sequester environmental metals,^77^^,^^78^ rendering them inaccessible to organisms lacking cognate siderophore receptors. Similarly, the diverse chemical structures of corrinoid cofactors dictate which bacteria can exploit which corrinoids via specific transporters and enzymes.^79^^,^^80^ Thus, OhyA activity reveals another example of the diverse mechanisms bacteria employ to competitively acquire, sequester, and utilize limited nutrients. Interestingly, OhyA products can also contribute to tolerogenic host signaling^56^ and inhibit other bacteria (as we show for *L. iners*) or fungi.^81^ Future studies will be required to elucidate how *Lactobacillus* OhyA enzymes mediate host-microbe and microbe-microbe interactions.

In addition to mechanistic insights, this study demonstrates therapeutic potential of OA and 10-HSA to improve BV treatment. Promoting *L. crispatus* over *L. iners* and more effectively targeting MTZ-resistant BV-associated bacteria are core goals for improving BV treatment.^29^^,^^30^^,^^31^ Our finding that OA and related molecules enhance *L. crispatus* growth while inhibiting *L. iners* and many BV-associated species—including MTZ-resistant strains of *Gardnerella* and *F. vaginae* (which have been linked to BV treatment failure^31^)—demonstrate OA’s potential to address this unmet clinical need. Current investigational therapies for BV include vaginal microbiome transplants and single- or multi-strain *Lactobacillus* live biotherapeutic products.^24^^,^^32^^,^^33^ However, efficacy has been modest in preliminary trials. We propose that combining topically applied OA and/or other uLCFA metabolites with MTZ, with or without a microbiome transplant or *L. crispatus*-containing live biotherapeutic, could improve treatment efficacy by promoting *L. crispatus* and inhibiting competition from other species.

In summary, we identify and mechanistically characterize important species-level differences in uLCFA resistance and metabolism among FGT *Lactobacillus* species, demonstrate their *in vivo* relevance, and provide preclinical evidence for a uLCFA-based therapeutic strategy for BV. This study illustrates how functional, metabolic, and genomic approaches can inform the development of microbiota-targeted therapies to improve human health.

### Limitations of the study

This study provides mechanistic and functional support for its primary conclusions but has several limitations. Bacterial genetic manipulation was performed only in *L. gasseri* due to lack of accessible genetic tools for other FGT *Lactobacillus* species—a long-standing challenge for the field.^82^^,^^83^^,^^84^^,^^85^^,^^86^ Conclusions about *farE* and *ohyA9* roles in *L. crispatus* are thus extrapolated from *L. gasseri*. Genetic tools were also lacking for heterologous expression of uLCFA-induced genes from non-*iners* lactobacilli in *L. iners*.^87^ Lack of animal models for human FGT *Lactobacillus* colonization and BV—another major challenge in the field^88^^,^^89^^,^^90^^,^^91^—necessitated use of an *in vitro* model of BV^36^ to assess OA and 10-HSA effects in community competition experiments. Human trials will thus be needed to fully assess uLCFA treatment effects on the FGT microbiota. Finally, we demonstrate therapeutic potential of exogenous uLCFAs. However, it remains to be determined how native FGT concentrations of uLCFAs may shape microbiome composition and whether factors such as diet, vaginal hygiene products, or other genetic or environmental factors influence FGT uLCFA concentrations in ways that affect *Lactobacillus* colonization.

## STAR★Methods

### Key resources table

**Table:** 

REAGENT or RESOURCE	SOURCE	IDENTIFIER
**Bacterial and virus strains**		

FGT bacterial isolate strains	See Table S1.	See Table S1.
EC1000, E. coli cloning host for pORI28 plasmids, RepA+	Addgene	71852
MC1061 Competent E. coli, E. coli cloning host for pTRK892 plasmids	Molecular Cloning Laboratories	MC1061
ATCC 33323, *L. gasseri* type strain	ATCC	33323
ATCC 4356, *L. acidophilus* type strain	ATCC	4356
ATCC 33323 with a clean gene deletion of LGAS_1245	This study	Wild-type (WT)
ATCC 33323 with an in-frame gene deletion of LGAS_1351	This study	ΔohyA9
ATCC 33323 with an in-frame gene deletion of LGAS_1630	This study	ΔfarE
ATCC 33323 with an in-frame gene deletion of LGAS_1351, complemented with pMZ12	This study	ΔohyA9/pohyA9
ATCC 33323 with an in-frame gene deletion of LGAS_1630, complemented with pMZ13	This study	ΔfarE/pfarE
Laboratory *S. aureus* strain that can accept DNA from *Escherichia coli*. It has a mutation in sau1 hsdR which makes it restriction deficient and a good intermediate cloning host.	BEI Resources	RN4220
USA300 with an in-frame gene deletion of SaohyA (PDJ68)	Subramanian et al.^49^	ΔSaohyA
USA300 with an in-frame gene deletion of SaohyA, complemented with empty expression vector (pPJ480)	Subramanian et al.^49^	ΔSaohyA/empty vector
USA300 with an in-frame gene deletion of SaohyA, complemented with SaohyA (pPJ490)	Subramanian et al.^49^	ΔSaohyA/pSaohyA
USA300 with an in-frame gene deletion of SaohyA, complemented with LCRIS_00661 (pLCRIS_00661)	This study	ΔSaohyA/pLCRIS_00661
USA300 with an in-frame gene deletion of SaohyA, complemented with LCRIS_00558 (pLCRIS_00558)	This study	ΔSaohyA/pLCRIS_00558

**Biological samples**		

Human specimens from FRESH, Durban, South Africa	See Table S2.	See Table S2.

**Chemicals, peptides, and recombinant proteins**		

90% Nitrogen, 5% Hydrogen, 5% Carbon dioxide gas	Airgas	X03NI90C3001054
Lactobacillus MRS Agar	Hardy Diagnostics	89407-144
Columbia Blood Agar (CBA)	Hardy Diagnostics	A16
BD DIFCO™ Lactobacilli MRS Broth	BD Biosciences	288130
L-Cysteine hydrochloride monohydrate	Millipore Sigma	C7880-100G
L-Glutamine	Millipore Sigma	G8540-100G
Proteose Peptone No. 3	BD Biosciences	211693
Yeast Extract Solution (100mL)	Gibco	18180-059
Gibco™ Horse Serum, Heat Inactivated, New Zealand Origin	Gibco	26050070
BHI Broth	BD Biosciences	211059
Fetal bovine serum (FBS), heat inactivated	Millipore Sigma	F4135
Vitamin K1-Hemin Solution	BD Biosciences	B12354
IsoVitaleX Enrichment	BD Biosciences	B11875
BD Difco-formulated Lysogeny broth	BD Biosciences	244610
BD Difco™ Dehydrated LB Agar	BD Biosciences	240110
≥98.5% Palmitoleic acid	Millipore Sigma	P9417
≥99% Oleic acid	Millipore Sigma	O1008
≥99% Linoleic acid	Millipore Sigma	L1012
Oleic acid-13C18	Millipore Sigma	490431
Oleic acid-13C18	Cambridge Isotope Laboratories, Inc.	CLM-460-PK
10-Hydroxystearic Acid, MS standard	Ambeed	A125712-50MG
10-Hydroxystearic Acid	Biosynth	FH175827
Sodium acetate	Millipore Sigma	S2889
Aqueous Glutaraldehyde EM Grade 8%	Electron Microscopy Services	16000
Paraformaldehyde, EM Grade, Purified Prill	Electron Microscopy Services	19200
Picric Acid Saturated Aqueous	Electron Microscopy Services	19552
Lysine hydrochloride	Electron Microscopy Services	L5626
Ruthenium Red, 37.5% Ru	Electron Microscopy Services	20600
Sodium Cacodylate Buffer, 0.2M, pH 7.4	Electron Microscopy Services	11650
Osmium Tetroxide	Electron Microscopy Services	19100
Propylene Oxide, Metal Can, EM Grade	Electron Microscopy Services	20401
Copper Parallel Bar Grids, 4-Quadrant	Electron Microscopy Services	G-GVPB-Cu
Lead Citrate 3%	Electron Microscopy Services	22410
Dextrose	VWR International LLC	BDH9230
Sodium Chloride	VWR International LLC	97063-368
Hepes	Sigma-Aldrich Inc	PHG0001
PBS (phosphate buffered saline)	Life Technologies Corporation	10010-049
Trizol	Life Technologies Corporation	15596026
FastPrep-24 5G	MP Biomedicals	116005500
SMARTScribe Reverse Transcriptase	Takara ClonTech	639538
Q5® High-Fidelity DNA Polymerase	New England Bio Labs	M0491S
BamHI-HF	New England Bio Labs	R3136S
SacI-HF	New England Bio Labs	R3156S
T4 DNA Ligase	New England Bio Labs	M0202
ZymoBroth	Zymo Research	M3015
S.O.C. medium	Invitrogen	15544034
QIAamp 96 DNA QIAcube HT kit	Qiagen Beverly LLC	51331
5-fluorouracil	Millipore Sigma	343922
Kanamycin sulfate	Millipore Sigma	K1377
Chloramphenicol	Millipore Sigma	220551
Erythromycin	Millipore Sigma	E5389
Metronidazole	Millipore Sigma	M1547

**Critical commercial assays**		

Cleanascite™ Lipid Removal Reagent and Clarification	Biotech Support Group	X2555-10
BD GasPak™ EZ Anaerobe Container System Sachets with Indicator	BD Biosciences	260001
(SPURR), Low Viscosity, Embedding Kit	Electron Microscopy Services	14300
0.1 mm Zirconia/Silica beads	BioSpec Products	11079101z
2 mL FastPrep tubes	MP Biomedicals	115065002
Direct-zol RNA Purification Kit	Zymo Research	R2070
Pan-Bacteria riboPOOL depletion kit	siTOOLs Biotech, Galen Laboratories	dp-K096
DNeasy Blood & Tissue Kit	Qiagen Beverly LLC	69504
QIAprep Spin Miniprep Kit	Qiagen Beverly LLC	27104
KAPA HiFi HotStart ReadyMix	Roche Holding AG	7958935001
E-Gel™ EX Agarose Gels, 2%	Invitrogen	G401002
QIAquick Gel Extraction Kit	Qiagen Beverly LLC	28704
UltraClean 96 PCR Cleanup Kit	Qiagen Beverly LLC	12596-4
Gibson Assembly® Master Mix	New England Bio Labs	T3001
Mix & Go! E.coli Transformation Kit	Zymo Research	M3015
0.1 mm glass beads	BioSpec Products	11079101
Whatman No. 1 filter paper	Millipore Sigma	WHA1001090
0.2 cm gap Gene Pulser Electroporation Cuvettes	Bio-rad Laboratories	1652086

**Deposited data**		

FGT Lactobacillus genome catalog	Bloom et al.^36^	https://doi.org/10.1038/s41564-022-01070-7
Completed Lactobacillus iners genomes	France et al.^34^	https://doi.org/10.1128/MRA.00234-20
Lactobacillaceae genome catalog	Zheng et al.^55^	https://doi.org/10.1099/ijsem.0.004107
FGT bacterial strain isolate genomes	This study	NCBI: PRJNA1127199
Lactobacillus bulk RNA sequencing	This study	GEO: GSE270628
16s rRNA sequencing from human vaginal swabs	This study	NCBI: PRJNA799634

**Oligonucleotides**		

Primers	See Table S4.	See Table S4.

**Recombinant DNA**		

pORI28	Addgene	71595
pTRK669	Addgene	71313
pTRK892	Addgene	71803
pMZ4: pORI28 derivative with homology arms the flanking LGAS_1245 (upp gene), the upstream and downstream arms are ∼600 bp	This study	pMZ4
pMZ7: pORI28 derivative with lacZ and upp gene inserted into the backbone	This study	pMZ7
pMZ9: pMZ7 derivative with homology arms internal to the LGAS_1351, the upstream and downstream arms are ∼600 bp	This study	pMZ9
pMZ10: pMZ7 derivative with homology arms internal to the LGAS_1630, the upstream and downstream arms are ∼600 bp	This study	pMZ10
pMZ12: pTRK892 derivative with the mutated gusA gene replaced by LGAS_1351	This study	pMZ12
pMZ13: pTRK892 derivative with the mutated gusA gene replaced by LGAS_1630	This study	pMZ13
pPJ480: S. aureus expression vector with a sarA promoter, empty vector	Subramanian et al.^49^	pPJ480
pPJ490: S. aureus expression vector with SaohyA gene under a sarA promoter	Subramanian et al.^49^	pPJ490
pLCRIS_00661: S. aureus expression vector with LCRIS_00661 gene under a sarA promoter	This study	pLCRIS_00661
pLCRIS_00558: S. aureus expression vector with LCRIS_00558 gene under a sarA promoter	This study	pLCRIS_00558

**Software and algorithms**		

Custom code	This study	Zenodo: https://zenodo.org/records/12615731
Adobe Illustrator	Adobe	www.adobe.com
DESeq2	https://doi.org/10.1186/s13059-014-0550-8	https://doi.org/10.18129/B9.bioc.DESeq2
QIIME I v1.9.188	https://doi.org/10.1038/s41587-019-0209-9	https://qiime2.org/
Dada2 v1.6.089	https://doi.org/10.1038/nmeth.3869	https://doi.org/10.18129/B9.bioc.dada2
Phlyoseq v1.30.090	https://doi.org/10.1371/journal.pone.0061217	https://doi.org/10.18129/B9.bioc.phyloseq
eggNOG 5.0	https://doi.org/10.1093/nar/gky1085	https://github.com/eggnogdb/eggnog-mapper
MUSCLE v5.1	https://doi.org/10.1038/s41467-022-34630-w	https://www.drive5.com/muscle/
raxmlGUI 2.0	https://doi.org/10.1111/2041-210X.13512	https://antonellilab.github.io/raxmlGUI/
FastTree v2.1	https://doi.org/10.1371/journal.pone.0009490	http://www.microbesonline.org/fasttree/#Install
Interactive Tree Of Life (iTOL) v5	https://doi.org/10.1093/nar/gkae268	https://itol.embl.de/
Python v3.9	Python	https://www.python.org/downloads/release/python-390/
Jupyter Notebook v6.5.2	Jupyter Notebook	https://jupyter-notebook.readthedocs.io/en/v6.5.2/
R v.3.6.3	The Comprehensive R Archive Network	https://cran.r-project.org/
R packages	N/A	seqinr v.4.2.5, tidyverse, v.1.3.1, knitr v.1.33, ggpubr v.0.4.0, DescTools v.0.99.41, gtools v.3.8.2, gridExtra v.2.3, cowplot v.1.1.1, scales v.1.1.1, grid v.3.6.3, broom v.0.7.6, e1071 v.1.7.6, and table1 v.1.4
Python packages	N/A	biopython v1.79, matplotlib v3.7.1, numpy v1.22.3, pandas v1.5.1, scikit-bio v0.5.8, scipy v1.9.3, seaborn v0.11.2, statannot v0.2.3, and statsmodels v0.13.2

**Other**		

Leica Reichert Ultracut-S microtome	Leica	N/A
JEOL 1200EX Transmission electron microscope	JEOL USA	N/A
AMT 2k CCD camera	Advanced Microscopy Techniques	N/A
Illumina NovaSeq SP 100	Illumina	N/A
NanoDrop 2000	Thermo Fisher Scientific	N/A
Gene Pulser Xcell Electroporation System	Bio-Rad	N/A
Leica Reichert Ultracut-S microtome	Leica	N/A
JEOL 1200EX Transmission electron microscope	JEOL USA	N/A
AMT 2k CCD camera	Advanced Microscopy Techniques	N/A
Illumina NovaSeq SP 100	Illumina	N/A
NanoDrop 2000	Thermo Fisher Scientific	N/A
Gene Pulser Xcell Electroporation System	Bio-Rad	N/A
Leica Reichert Ultracut-S microtome	Leica	N/A

### Resource availability

#### Lead contact

Further information and requests for resources and reagents should be directed to and will be fulfilled by the lead contact, Douglas S. Kwon (dkwon@mgh.harvard.edu).

#### Materials availability

All unique/stable reagents directly used in this study are available from the lead contact with a completed Materials Transfer Agreement. The empty vector, pMZ7, used for double homologous recombination (Addgene #223180) is publicly available. The *upp* gene-deleted mutant strain of *L. gasseri* used as a parent strain for constructing gene knockouts (Δ1245-WT) has been submitted to NIAID for consideration to include in the BEI Resources program.

#### Data and code availability

• Long-read sequence genomes (NCBI BioProject ID: PRJNA1127199), bulk RNA-sequencing data (GEO: GSE270628), and 16s rRNA gene sequencing data (NCBI BioProject ID: PRJNA799634, sequence accession details for individual samples listed in Table S2) from human vaginal swab samples are publicly available and accessible.
• All original code for transcriptomic analysis, bacterial genomic analysis, human *h*FA analysis, and bacterial *in vitro* competition assay analysis is deposited at Zenodo and publicly available and accessible under https://zenodo.org/records/12615731.
• Any additional information required to reanalyze the data reported in this work paper is available from the lead contact upon request.

### Experimental model and study participant details

#### Bacteria strains and culture conditions

Bacterial strains used in this study for each experiment/figure are summarized in Table S1. All vaginal bacterial isolates were cultured under anaerobic conditions at 37–40°C using a palladium catalyst-based anaerobic chamber (COY) with an atmosphere of 5% carbon dioxide, 5% hydrogen, and 90% nitrogen (Airgas #X03NI90C3001054). All media, culture reagents, and plastic-ware were pre-reduced by placement overnight in the anaerobic chamber before use. Vaginal bacterial isolates were first revived on solid agar plates to obtain single colonies. After 4–5 days of incubation, representative colonies were picked into liquid culture. All *Lactobacillus* species isolates were revived from frozen 25% glycerol stocks on MRS agar plates (Hardy Diagnostics #89407-144), except for *Lactobacillus iners*, which was revived on Columbia Blood Agar (CBA) agar plates (Hardy Diagnostics #A16). Non-*Lactobacillus* FGT species were revived from frozen 25% glycerol stocks on CBA agar plates. For cultivation of *Lactobacillus* species in liquid media, modified Lactobacillus MRS (MRS+CQ) broth in static culture was used unless otherwise noted. For cultivation of non-*Lactobacillus* species, NYCIII broth in static culture was used unless otherwise noted.

ATCC 33323 *Lactobacillus gasseri* and derivative mutant strains were cultured under anaerobic conditions for solid media and aerobic conditions for liquid media at 37°C. Strains were revived from frozen 25% glycerol stocks on MRS agar plates for 48 hours under anaerobic conditions using an anaerobic box with oxygen-eliminating sachets padded with a methylene blue anaerobic indicator (BD GasPak™ EZ Anaerobe Container System Sachets with Indicator #260001). After incubation, a single colony was picked into liquid culture and incubated aerobically at 37°C with shaking. MRS+CQ broth was used for liquid culture unless otherwise noted.

*Escherichia coli* cloning strains, EC1000 (Addgene #71852) and MC1061 (Molecular Cloning Laboratories #MC1061), *S. aureus*, and *S. aureus* derivative mutant strains were cultured under aerobic conditions at 37°C with shaking for liquid cultures. EC1000 was revived from frozen 25% glycerol stocks on LB agar plates supplemented with 40 μg/mL kanamycin (KAN). After overnight incubation, a single colony was picked into liquid culture and incubated aerobically at 37°C with shaking. LB media supplemented with 40 μg/mL KAN was used for liquid culture unless otherwise noted. MC1061 strains with a pTRK892-derived plasmid (Addgene #71803) were revived from frozen 25% glycerol stocks on LB agar plates supplemented with 200 μg/mL erythromycin (ERY). LB media supplemented with 200 μg/mL ERY was used for liquid culture.

Media additives were added to autoclave-sterilized broth or agar plates after cooling to room temperature. Broth was re-sterilized using a 0.22 μM PES filter after all additives were added. To prepare MRS+CQ broth, BD Difco-formulated *Lactobacillus* MRS (De Man, Rogosa and Sharpe) broth (BD Biosciences #288130) was prepared as per the manufacturer’s instructions and then supplemented with L-cysteine (4 mM) and *L*. glutamine (1.1 mM) as previously described.^36^ To prepare lipid-depleted MRS+CQ broth, four parts MRS+CQ broth was combined with one part Cleanascite™ Lipid Removal Reagent (Biotech Support Group #X2555-10) and shaken (220 rpm) at room temperature for 10 min, then centrifuged at 2,000 x g for 15 min. The media supernatant was collected and re-sterilized using a 0.22 uM PES filter. NYCIII broth (ATCC medium 1685) pre-media was prepared with 15 g/L Proteose Peptone No. 3 (BD Biosciences #211693), 5 g/L sodium chloride, and 4 g/L HEPES in 875 mL of distilled water, pH-adjusted to 7.3, and autoclaved. After autoclaving and cooling the pre-media, dextrose solution (3 g / 45 mL distilled water) was added at 7.5% v/v, Gibco yeast extract solution (Gibco #18180-059) was added at 2.5% v/v, and heat inactivated horse serum (Gibco #26050070) was added at 10% v/v. The complete NYCIII broth was then sterilized by passage through a 0.22 μm PES filter. S-broth^36^ pre-media was prepared with 37 g/L Brian Heart Infusion (BHI) broth (BD Biosciences #211059), 10 g/L yeast extract powder, and 1 g/L dextrose in 880 mL distilled water, brought to a boil until dissolved, and then autoclaved. After autoclaving and cooling the pre-media, fetal bovine serum (Millipore Sigma #F4135) was added at 5% v/v, vitamin K1-Hemin solution (BD Biosciences #B12354) was added at 5% v/v, and IsoVitaleX (BD Biosciences #B11875) enrichment was added at 2% v/v. The complete S-broth was then sterilized by passage through a 0.22 μm PES filter. To prepare LB media and agar, BD Difco-formulated Lysogeny broth (LB) media (BD Biosciences #244610) or agar (BD Biosciences #240110) plates were prepared as per the manufacturer’s instructions and then supplemented with pre-sterilized stocks of antibiotics or sodium acetate where indicated. For all fatty acid supplementations, oleic acid (≥99% purity Millipore Sigma #O1008), universally ^13^C_18_-labeled oleic acid (^13^C_18_-OA; ≥99% purity, Millipore Sigma #490431), palmitoleic acid (≥98.5% purity, Millipore Sigma #P9417), and linoleic acid (≥99% purity, Millipore Sigma #L1012) were directly added to broth media.

#### FRESH cohort and human samples

The FRESH cohort is an ongoing prospective observational study based in Umlazi, South Africa, that enrolls 18–23 year old, HIV-uninfected, sexually active, healthy women able to understand and provide written informed consent. Study characteristics and inclusion and exclusion criteria are also described elsewhere.^10^^,^^13^^,^^36^^,^^92^ In summary, participants had to be willing to adhere to study requirements and have HIV tests performed two times a week along with other vaginal swab and cervical samples collected and stored. Exclusion criteria included pregnancy, anemia, any chronic medical condition or other conflict likely to prevent study protocol adherence, and/or enrollment in any other study that involves frequent blood sampling or that might otherwise interfere with the FRESH study protocol. The study protocol was approved by the Massachusetts General Hospital (MGH) Institutional Review Board (IRB, 2012P001812/ MGH) and the Biomedical Research Ethics Committee of the University of KwaZulu-Natal (UKZN; Ethics Reference Number BF131/11). All participants provided written informed consent.

Twice per week at the study site, participants received HIV RNA viral load testing by PCR and attended classes focused on personal empowerment, job skills training, and HIV prevention. Once every 12 weeks, participants provided peripheral blood and mucosal specimens described in more detail below. Participants were additionally provided a light meal during study site visits and cumulative monetary compensation over a 36-week period of ZAR3,700 (∼US$280), meant to help defray transportation expenses for the twice-weekly study site visits.

For FRESH study mucosal specimen collections, swab samples (Puritan 6” sterile standard foam swab with polystyrene handle) were collected by swabbing the posterior fornix or by swabbing the mid-vaginal wall under direct visualization during speculum exam. The posterior fornix swabs were used to make a slide preparation for Gram stain analysis and the vaginal swabs werer frozen without cryopreservatives for nucleic acid extraction and microbiota profiling. Cervicovaginal lavage (CVL) samples were collected using a flexible plastic bulb pipette to dispense 5 ml of sterile normal saline into the vagina and wash the cervix four times. Fluid was then re-aspirated into a 15 ml conical tube. CVL and vaginal swab samples were stored on ice for 1–4 hours during transport to the processing laboratory at the HIV Pathogenesis Programme, Doris Duke Medical Research Institute at the Nelson R. Mandela School of Medicine at UKZN, where swabs were stored at –80°C. CVL samples were centrifuged (700xg, 10 min at 4°C). Supernatants from the centrifuged CVL samples were then transferred to cryovials and stored at –80°C.

### Method details

#### Bacterial culture conditions for growth inhibition, growth enhancement, and killing assays

To assess mono-culture growth effects of various media formulations and additives, bacterial isolate stocks were first revived on solid media for 2–5 days until fully formed colonies were present. Starter liquid cultures were then inoculated with a few representative colonies for each strain and grown for 48 hours. After 48 hours, the optical density at 600 nm (OD600) of each liquid mono-culture was measured and each cultivated strain was back-diluted to 40X the starting OD600 (e.g. 40X OD600=0.8 for a starting OD600=0.02) for each experiment using the base media specific to that experiment. In cases where growth enhancement was being assessed, starter cultures were pelleted (5,000xg, 5 min at RT), washed with phosphate-buffered saline (PBS) three times, resuspended in PBS, and back-diluted to 40X the starting OD600 prior to assay inoculation to prevent nutrient carryover. Back-diluted starter cultures were used to inoculate the experimental media conditions. At the selected time point for each experiment, growth was assessed by optical density at 600 nm (OD600) with a SpectraMax M5 (Molecular Devices).

To assess bactericidal activity of OA, a minimum bactericidal concentration (MBC) assay was performed in MRS+CQ broth with varying concentrations of OA. Starter cultures and experimental conditions were prepared as described above. After 24 hours of incubation, colony forming units (CFU) were measured by serially diluting each culture condition 10-fold (e.g. 10 μL of culture into 100 μL of media) and 10 μL of each dilution was plated from neat (undiluted) to 10^-7^ dilution. Colonies were counted manually after 48–72 hours of incubation.

#### Competition and mock community culture experiments

Starter cultures of representative strains of *L. crispatus*, *L. gasseri*, *L. iners*, *L. jensenii*, *G. piotii*, *G. vaginalis*, *P. amnii*, *P. bivia*, *P. disiens*, *P. timonensis*, *F. vaginae*, and *S. vaginalis* were prepared in NYCIII broth. Aliquots of mono-cultured strains were mixed in defined ratios to create defined, mock communities. CFU inputs for each strain relative to *L. crispatus 1* were as follows in Figures 7B, 7C, S7A, and S7B: *L. crispatus 2*, 0.7; *L. gasseri*, 6; *L. jensenii*, 0.08; *L. iners 1*, 8; *L. iners 2*, 20; *G. piotii*, 60; *G. vaginalis*, 32; *P. amnii*, 20; *P. bivia*, 8; *P. disiens*, 70; *P. timonensis*, 30; *F. vaginae*, 11; *S. vaginalis*, 3. CFU inputs for each strain relative to *L. crispatus 1* were as follows in Figures 7D, 7E, and S7C: *L. iners*, 12; *G. vaginalis*, 17; *P. amnii*, 11; *P. bivia*, 22; *F. vaginae*, 10; *S. vaginalis*, 18. Communities were then divided and used to inoculate replicate cultures across different treatment conditions for incubation (6 replicates per condition) by adding 150 μL of mixture in V-bottom 96 well plates. Treatment conditions included media (NYCIII or S-broth) with no supplementation, media supplemented with 50 μg/mL metronidazole, media supplemented with 3.2 mM oleic acid, media supplemented with 1.6 mM 10-HSA, media supplemented with both 3.2 mM oleic acid and 50 μg/mL metronidazole, or media supplemented with both 1.6 mM 10-HSA and 50 μg/mL metronidazole. CFU (colony forming unit) titres were determined for each axenic culture as described above and used to calculate starting ratios within the mixed cultures. At 48 or 72 hours (and indicated in figure legends), cultures were harvested by centrifuging at 5,000xg for 10 min at 4°C, supernatant was removed, and pellets were frozen at –20°C for subsequent DNA extraction and analysis. For the community passaging experiment, 2.5% v/v of the treated communities were passaged into fresh NYCIII broth (without OA or MTZ) prior to centrifugation and freezing of the remainder of the culture, then cultured for another 48 hours, which was then repeated for a second passage. Relative growth within mixed cultures was assessed by bacterial 16S rRNA gene sequencing as described below. OD600 was determined for each input community mixture (t=0 hours) and the final time point (t=72 hours) in the no-treatment control condition to confirm growth of the defined, *in vitro* community cultures. Aliquots of the mono-cultured strains were assayed separately in all treatment conditions to confirm expected growth patterns, and assessed by bacterial 16S rRNA gene sequencing at the corresponding time points to confirm purity.

#### Sample preparation and conditions for TEM imaging

For TEM sample preparation, representative strains of *L. crispatus* and *L. iners* were revived on solid media, representative colonies were picked into liquid cultures, and incubated for 48 hours under standard culture conditions. Cultures were back-diluted to a starting OD600 of 0.02 in untreated media and grown to exponential phase, OD600=0.4–0.6. Exponential cultures were then either left untreated or treated with 3.2 mM OA for 1 hour. After fatty acid treatment, cultures were removed from the anaerobic chamber, combined in a 1:1 ratio with fixative solution prepared at 2X the working concentration, and promptly pelleted (5,000xg, 5 min at RT). Samples were kept at room temperature for 2 hours to allow for pellet fixation. Fixative solution at 2X the working concentration contained 5% Glutaraldehyde (Electron Microscopy Services (EMS) #16000), 2.5% Paraformaldehyde (EMS #19200), 0.06% picric acid (EMS #19552), 20 mM Lysine (EMS #L5626), and 0.2% Ruthenium Red (EMS #20600) in 0.2 M cacodylate buffer pH 7.4.

Fixed pellets were washed three times in 0.1% Ruthenium Red in 0.1 M cacodylate buffer (EMS #11650) and postfixed with 1% Osmium tetroxide (OsO4, EMS #19100) + 0.1% Ruthenium Red for 2 hours, washed two times in water + 0.1% Ruthenium Red, and subsequently dehydrated in grades of ethanol (10 min each; 50%, 70%, and 90% ethanol once, and 100% ethanol twice). The samples were then put in propylene oxide (EMS #20401) for 1 hour and infiltrated overnight in a 1:1 mixture of propylene oxide and Spurr’s Low Viscosity Embedding media (EMS Catalog #14300). The following day the samples were embedded in Spurr’s Low Viscosity Embedding media (EMS #14300) and polymerized at 60°C for 48 hours. Ultrathin sections (about 80 nm) were cut on a Reichert Ultracut-S microtome, picked up on to copper grids (EMS #G-GVPB-Cu) stained with lead citrate (EMS 22410) and examined in a JEOL 1200EX Transmission electron microscope and images were recorded with an AMT 2k CCD camera.

#### ATP release assays

For the ATP release assays, representative strains of *L. crispatus*, *L. iners*, *P. bivia*, *P. timonensis*, and the *L. gasseri* wild-type, *ΔfarE*, and *ΔfarE/pfarE* strains were revived on solid media, representative colonies were picked into liquid cultures (MRS+CQ broth for *Lactobacillus* species and NYCIII broth for *Prevotella* species), and incubated for 24 or 48 hours under standard culture conditions. Cultures were back-diluted to a starting OD600 of 0.02 in untreated media and grown to mid- to late-exponential phase, OD600=0.4–0.6. Exponential cultures were then either left untreated or treated with varying concentrations of OA for 0, 30, 60, or 120 min. After treatment, cultures were removed from the anaerobic chamber and promptly pelleted (5,000xg, 5 min at RT). Supernatants and pellets were collected and each stored separately at –20°C for subsequent ATP quantification. The concentration of ATP in each sample was quantified using the BacTiter-Glo assay reagent kit (Promega #G8230) for each supernatant and pellet sample. In brief, 25 μL of the BacTiter-Glo reagent was added to 25 μL of each sample in a white opaque 384-well plate, shaken at RT for 5 min, and luminescence was readout using a plate reader with a 250 ms integration time. The relative ATP release was calculated by dividing the concentration of ATP detected in the supernatant by the concentration of ATP detected in the sample’s corresponding pellet and expressed relative to the ATP release measured at 0 min with no OA treatment (T0 sample).

#### Sample preparation for bulk RNA-sequencing of bacterial isolates

For transcriptomic profiling of *cis*-9-uLCFA-treated non-*iners Lactobacillus* cultures, representative strains of *L. crispatus*, *L. gasseri*, and *L. jensenii* were revived on solid media, then representative colonies were picked into liquid cultures and incubated for 48 hours under standard culture conditions. Cultures were back-diluted to a starting OD600 of 0.02 in untreated media and grown to exponential phase, OD600=0.4–0.6. Exponential cultures were then either left untreated or treated with OA, LOA, or POA (3.2 mM each). To collect samples for bulk RNA sequencing after 1 hour of treatment, cultures were pelleted (5,000xg, 5 min at 4°C), the supernatant removed, and the pellets were resuspended in 500 μL of Trizol (Life Technologies Corporation #15596026), immediately put on dry ice, and stored at –80°C until RNA extraction and library preparation. Three replicates, each from an independent starter culture, were included for each strain and condition.

#### RNA extraction from bacterial isolates

Trizol-preserved bacterial samples prepared as above were thawed, transferred to 2 mL FastPrep tubes (MP Biomedicals #115065002) containing 500 μL of 0.1 mm Zirconia/Silica beads (BioSpec Products #11079101z), and bead beaten for 90 seconds at 10 m/sec speed using the FastPrep-24 5G (MP Biomedicals #116005500) with a Metal QuickPrep adapter. After bead beating, samples were incubated on ice for 3 min, and 200 μL chloroform was added to each sample and mixed by tube inversion. After 3 min incubation at room temperature, samples were centrifuged (12,000xg, 15 min at 4°C) to form separated phase layers between the organic & aqueous sections. For each sample, 200 μL of the clear aqueous phase was transferred to separate clean tubes, mixed with an equal volume (200 μL) of 100% ethanol by tube inversion, and incubated for 5 min at RT. Using a Direct-zol RNA Purification kit (Zymo Research #R2070), each sample was then transferred to a Direct-zol spin column and centrifuged (16,500xg, 1 min at RT). Columns were washed twice with 400 μL Direct-zol RNA Pre-Wash Buffer, and once with 700 μL Direct-zol RNA Wash Buffer and then centrifuged at max speed for 2 min. To dry the pellet and remove any ethanol carryover, columns were centrifuged with lids open (12,000xg, 1 min at RT). Finally, columns were moved to RNase-free 1.5mL tubes, incubated with 100 μL of nuclease-free water for 5 min, and then eluted by centrifugation (12,000xg, 1 min at RT). Extracted RNA samples were kept on ice for use and QC or stored at –80°C.

#### Library preparation for bulk RNA-sequencing of bacterial isolates

Illumina cDNA libraries were generated using a modified version of the RNAtag-seq protocol.^93^ Briefly, 250 ng of total RNA was fragmented, depleted of genomic DNA, dephosphorylated, and ligated to DNA adapters carrying 5’-AN_8_-3’ barcodes of known sequence with a 5’ phosphate and a 3’ blocking group (IDT). Barcoded RNA molecules were pooled and depleted of rRNA using the Pan-Bacteria riboPOOL depletion kit (siTOOLs Biotech, Galen Laboratories #dp-K096). Pools of barcoded RNAs were converted to Illumina cDNA libraries in 2 main steps: (i) reverse transcription of the RNA using a primer designed to the constant region of the barcoded adaptor with addition of an adapter to the 3’ end of the cDNA by template switching using SMARTScribe Reverse Transcriptase (Takara ClonTech #639538) as described^94^; (ii) PCR amplification using primers whose 5’ ends target the constant regions of the 3’ or 5’ adaptors and whose 3’ ends contain the full Illumina P5 or P7 sequences. cDNA libraries were sequenced on the Illumina NovaSeq SP 100 platform to generate paired end reads.

#### Analysis of RNA-sequencing data

Since samples were barcoded in library preparation and then pooled for sequencing, reads from each sample were demultiplexed based on their associated barcode sequence using custom scripts. Up to 1 mismatch in the barcode was allowed, provided it did not assign the read a separate barcode included in the sequencing pool. Barcode sequences were trimmed from the first read, as were terminal G’s from the second read that may have been added by SMARTScribe during template switching.

For each sample, reads were aligned to the sample species reference genome (GCF_022455535.1 / ASM2245553v1 for *L. crispatus*; GCF_022456925.1 / ASM2245692v1 for *L. gasseri*; GCF_022456915.1 / ASM2245691v1 for *L. jensenii*) using BWA^95^ and read counts were assigned to genes and other genomic features using custom scripts. Differential expression analysis was conducted using DESeq2.^46^ The Benjamini-Hochberg procedure was used to control the false discovery rate (FDR) with ɑ=0.05.

#### Preparation of spent media and pellets from cultured bacteria for untargeted lipidomics and isotopic tracing

Starter cultures were prepared as described above, pelleted, and washed 3 times with PBS. Washed pellets were resuspended in PBS using the same volume as the initial start culture. Washed starter cultures were then back-diluted into 0, 0.1, or 3.2 mM unlabeled OA or ^13^C_18_-OA and allowed to incubate under standard culture conditions for 24 or 72 hours as indicated. After incubation, cultures were pelleted (5,000xg, 10 min at 4°C). Supernatants were removed, collected into separate tubes, and placed onto dry ice until storage at –80°C. Cell pellets were washed once with ice cold PBS. Washed pellets were also placed onto dry ice and then stored at –80°C once frozen. Three replicates were included for each strain and condition.

#### Exogenous OA accumulation assays

*L. gasseri ΔfarE* and *ΔfarE/pfarE* strains were revived on solid media, representative colonies were picked into liquid cultures, and incubated for 24 hours under standard culture conditions. Cultures were back-diluted to a starting OD600 of 0.02 in untreated media and grown to mid- to late-exponential phase, OD600=0.4–0.6. Exponential cultures were then either left untreated or treated with 100 or 400 μM ^13^C_18_-OA for 15 min in NYCIII broth. After treatment, cultures were removed from the anaerobic chamber and promptly pelleted (5,000xg, 5 min at RT). Cell pellets were washed once with ice cold PBS. Washed pellets were placed onto dry ice and then stored at –80°C once frozen. Five replicates were included for each strain and condition.

#### Plasmid construction methods for strain generation in *L. gasseri* ATCC 33323

All vectors and primers with their respective annealing temperatures are noted in the key resources table and Table S4, respectively. Primers were purchased/synthesized by IDT. All cloning host strains and generated mutant strains are noted in the Reagents Table. All plasmids were verified with nanopore-based whole plasmid sequencing using Primordium Labs services. All mutant strains were verified with Illumina-based whole-genome sequencing (WGS) using SeqCenter, LLC.

Genomic DNA extractions were performed using the DNeasy Blood & Tissue Kit (Qiagen Beverly LLC #69504) following manufacturer protocols for Gram positive bacterial pellet samples. Minipreps were performed using the QIAprep Spin Miniprep Kit (Qiagen Beverly LLC #27104) following the manufacturer’s protocol. Gel extractions were performed using the QIAquick Gel Extraction Kit (Qiagen Beverly LLC #28704) following the manufacturer’s protocol. Extracted and amplified DNA products were quantified using a NanoDrop 2000. DNA bands were visualized using E-Gel™ EX Agarose Gels (2%, Invitrogen #G401002) under UV light.

Q5® High-Fidelity DNA Polymerase (New England Bio Labs (NEB) #M0491S) was used for PCR amplification of products to be used for plasmid construction following the manufacturer’s protocol. Briefly, Q5® High-Fidelity DNA Polymerase PCR reactions were performed in 25 μL reactions such that the final concentration of reagents were as follows, 1X Q5 Reaction Buffer, 200 μM dNTPs, 0.5 μM Forward Primer, 0.5 μM Reverse Primer, 0.02 units/μL Q5 High-Fidelity DNA Polymerase, and 1–10 ng of template DNA; and, thermocycling was performed at 98°C for 30 s, followed by 30 cycles of 98°C for 10 s, annealing temperature (noted in Table S4 for each primer pair) for 30 s, and 72°C for 20 s/kb of desired amplicon, with a final 2 min extension at 72°C. KAPA HiFi HotStart ReadyMix (Roche Holding AG #07958935001) was used for all colony PCR-screening reactions following the manufacturer’s protocol. KAPA HiFi HotStart ReadyMix PCR reactions were performed in 25 μL reactions such that the final concentration of reagents were as follows, 1X Ready Mix, 0.3 μM Forward Primer, 0.3 μM Reverse Primer, and a pipette-tip sample of a single colony; and, thermocycling was performed at 95°C for 3 min, followed by 35 cycles of 98°C for 20 s, annealing temperature (noted in Table S4 for each primer pair) for 15 s, and 72°C for 20 s/kb of desired amplicon, with a final 1 min extension at 72°C.

For restriction and ligation-based cloning, restriction enzyme-based digestion was performed at 37°C for 15 min following manufacturer’s protocols. Digestion reactions were performed in 50 μL reactions such that the final concentration of reagents were as follows, 1X rCutSmart Buffer and 0.4 units/μL per enzyme. Ligation was performed at room temperature for 10 min and then heat inactivated at 65°C for 10 min. Reactions were performed in 20 μL reactions such that the final concentration of reagents were as follows, 1X T4 DNA Ligase Buffer, 37.5 ng of insert fragment, 50 ng of linearized backbone, and 1 unit/μL of T4 DNA Ligase (NEB #M0202). A no-insert with backbone only control was prepared in parallel with each ligation reaction to serve as a backbone self-ligation control. For Gibson cloning, Gibson Assembly® Master Mix (NEB #E2611) was used for all Gibson assembly reactions following the manufacturer’s protocol. Gibson assemblies were performed in 20 μL reactions such that the final concentration of reagents were as follows, 1X Gibson Assembly Master Mix with 0.02–0.5 pmols of DNA fragments comprising 3–5 parts per insert fragment and 1 part linearized backbone. For plasmid construction, *E. coli* EC1000 strain^96^ (Addgene #71852), a kanamycin resistant strain carrying a single copy of the repA gene in the glgB gene, was used as the cloning host for all pORI28-based plasmids, and *E. coli* MC1061 strain (Molecular Cloning Laboratories #MC1061) was used as the cloning host for all pTRK892-based plasmids.

#### Plasmid transformations methods for strain generation in *L. gasseri* ATCC 33323

Competent cells of *E. coli* EC1000 strain were prepared using the Mix & Go! *E. coli* Transformation Kit (Zymo Research #T3001) following the manufacturer’s protocol. 0.5 mL of an overnight culture of EC1000 grown in LB media at 37°C was transferred to 50 mL of ZymoBroth (Zymo Research #M3015) and cultured for 15–16 hours at 22°C with shaking to obtain a culture of OD600=0.4. Wash and Competent Buffers were diluted to 1X working concentration using the provided Dilution Buffer and kept on ice. The grown ZymoBroth culture was then pelleted (5,000xg, 10 min at 4°C), supernatant was removed, and pellets were washed with 5 mL of 1X Wash Buffer. The washed pellets were gently resuspended in 5 mL of ice cold 1X Competent Buffer and aliquoted into 100 μL volumes on ice. Aliquots were immediately used or stored at –80°C for transformation at a later time. For transformation of competent EC1000, 5 μL of plasmid DNA was added to 100 μL of cells, gently mixed, and incubated for 10 min on ice. After incubation, 400 μL of S.O.C. medium (Invitrogen #15544034) was added to the cells, which were then incubated for 1 hour at 37°C with shaking, plated onto pre-warmed LB agar with antibiotic selection, and incubated overnight at 37°C. The no-insert cloning control and no DNA plasmid control samples were transformed and plated in parallel as negative controls for each transformation.

For transformation of *E. coli* MC1061, competent MC1061 were thawed on ice, aliquoted into 100 μL volumes into chilled, clean 1.5 mL microcentrifuge tubes, gently mixed with 5 μL of plasmid DNA, and incubated for 15 min on ice. Cells were heat-shocked for 45 seconds in a 42°C water bath and then immediately placed on ice for 2 min. 0.9 mL of S.O.C. medium was added, cells were incubated for 1 hour at 37°C with shaking, plated onto pre-warmed LB agar with antibiotic selection, and incubated overnight at 37°C. The no-insert cloning control and no DNA plasmid control samples were transformed and plated in parallel as negative controls for each transformation.

Competent cells of *L. gasseri* ATCC 33323 and derivative mutants were prepared fresh for each transformation. 3.5X sucrose:MgCl2 electroporation buffer (3.5X SMEB) buffer was used as the transformation buffer and prepared such that the final concentration was 952 mM sucrose and 3.5 mM MgCl2 at pH 7.2 in DI water and then sterilized using a 0.22 uM PES filter. A 500 mL stock of 3.5X SMEB was prepared fresh each month and stored at 4°C. A single colony of *L. gasseri* WT or mutant was picked and cultured aerobically in 100 mL of MRS broth for 15–16 hours at 37°C with shaking. After 15–16 hours, cells were pelleted (5,000xg, 10 min at 4°C) and resuspended on ice with 100 mL of ice cold 3.5X SMEB buffer. The resuspension was carefully mixed until the pellet was completely resuspended into a homogenous solution, re-pelleted (5,000xg, 10 min at 4°C), and then resuspended on ice again in 5 mL of ice cold 3.5X SMEB to obtain the electrocompetent cell mixture. 100 μL of the electrocompetent cells were aliquoted into pre-chilled 0.2 cm gap Gene Pulser Electroporation Cuvettes (Bio-rad Laboratories #1652086) and kept on ice. 1 ug of DNA plasmid (volume kept < 10 μL to prevent sparking) was added to the cuvette, gently mixed without creating any air bubbles, and incubated on ice for 5 min. After incubation, electroporation was performed with a Gene Pulser Xcell Electroporation System using the following conditions: 1.25 kV, 25 μF, and 200 Ω. Immediately after electroporation, the cuvette was held on ice for 5 min, then the sample was added to 1 mL of pre-warmed MRS broth and incubated for 3 hours at 37°C with shaking. After incubation, cells were gently pelleted (300xg, 5 min), plated onto MRS agar plates with selection antibiotic, and incubated for 48 hours at 37°C in an anaerobic box.

#### Gene knockout and complementation in *L. gasseri* ATCC 33323

*L. gasseri* ATCC 33323 was selected as a genetically tractable, representative non-*iners Lactobacillus* strain exhibiting *cis-*9-uLCFA resistance and OA growth enhancement phenotypes similar to other strains of *L. gasseri*, *L. crispatus*, *L. jensenii*, and *L. mulieris*. To generate gene knockout mutants in *L. gasseri* ATCC 33323, we adapted a previously reported uracil phosphoribosyltransferase (*upp*)-based two-plasmid homologous recombination system.^60^^,^^61^^,^^97^ This system exploits the 5-fluorouracil (5-FU) resistance of a *upp*-deficient parent strain for knockout construction. To briefly summarize the underlying principles, uracil phosphoribosyltransferase, central to the pyrimidine salvage pathway, catalyzes the conversion of uracil to uridine monophosphate. When provided 5-FU, uracil phosphoribosyltransferase will produce 5-fluorouridine-5′-monophosphate, which is a suicide inhibitor to thymidylate synthase, a required enzyme in DNA synthesis. Vectors used to generate the *upp*-deficient strain and other gene deletion mutants included pTRK669,^61^ pORI28,^96^ and pORI28-derived plasmids. pORI28 is an empty backbone that requires RepA for stable plasmid replication and propagation and that encodes an erythromycin (ERY) resistance gene as a selectable marker; it is used for chromosomal integration in Gram positive bacteria. pMZ7 is a pORI28-derived backbone inserted with *lacZ* and the *L. acidophilus upp* gene (*Laupp*), which serves as the counterselection gene when integrated chromosomally. pTRK669 is a chloramphenicol (CHLOR) resistant, temperature sensitive helper plasmid that encodes RepA. To summarize the overall approach, we first generated an *upp* gene-deleted mutant in *L. gasseri* ATCC 33323 (Δ1245-WT; generated as described below), which was resistant to 5-FU. This Δ1245-WT mutant served as the parent strain for all additional mutants and is therefore referred to as the WT strain in the figures and the results and discussion text. The pTRK669 helper plasmid was electro-transformed into Δ1245-WT to create Δ1245-WT/pTRK669. Then pMZ7-derived vectors containing ∼600-bp homology arms within each gene-of-interest were constructed and respectively electro-transformed into Δ1245-WT/pTRK669, where RepA expression from pTRK669 enabled them to be replicated and propagated. Δ1245-WT/pTRK669 strains containing each gene-of-interest-specific pMZ7-derived vector were cultured under ERY and CHLOR selection (7.5 μg/mL CHLOR and 5 μg/mL ERY) at 37°C, then subcultured 3–5 times under ERY selection only (2.5 μg/mL) at 42°C to cure the pTRK669 helper plasmid and select for single-crossover chromosomal integrants of the pMZ7-derived vector. After these subculturing steps, cells were plated onto MRS agar plates with ERY (5 μg/mL) and colony PCR-screened for pMZ7-derived vector chromosomal integration (primers used to screen individual mutants are described below). Confirmed clones of Δ1245-WT containing single-crossover integrants of the pMZ7-derived vector targeting the gene-of-interest were next cultured without antibiotic selection to allow for vector resolution from the chromosome, then subcultured in 5-FU to counter-select for the desired gene deletion mutant strain via a second crossover event to generate an in-frame gene deletion knockout by double homologous recombination. All subculturing steps were performed by transferring 5% of the grown culture volume into the newly prepared broth of equal volume to make a 5% inoculum.

We generated the Δ1245-WT parent strain used for the above knockout approach as follows. To construct the pMZ4 vector for generating Δ1245-WT, we generated a pORI28 derivative with homology arms flanking the endogenous *upp* gene (LGAS_1245), each being ∼600 bp for the upstream and downstream arms, cloned into pORI28 using restriction digest and ligation cloning methods. In brief, a 594-bp upstream region and a 616-bp downstream region flanking LGAS_1245 were each amplified from genomic DNA extracted from *L. gasseri* ATCC 33323 to produce PCR amplicons, LGAS_1245-up and LGAS_1245-dwn, (primers for LGAS_1245-up: 1245-SOE-1 and 1245-SOE-2; primers for LGAS_1245-dwn: 1245-SOE-3 and 1245-SOE-4). Splicing by overlap-extension PCR was used to fuse LGAS_1245-up and LGAS_1245-dwn, producing a single 1187-bp amplicon (primers: 1245-SOE-1 and 1245-SOE-4), which was gel-purified to yield only the fused amplicon product. The fused LGAS_1245 homology arm amplicon and pORI28 were each digested separately using BamHI-HF and SacI-HF. The digested pORI28 backbone was additionally treated with shrimp Alkaline Phosphatase (rSAP) during the digest reaction and gel-purified before use in cloning. The digested pORI28 backbone and fused LGAS_1245 homology arm amplicon were ligated using T4 ligase and subsequently deactivated by heating. 10 μL of the ligation reaction was used to transform competent EC1000 cells, which were then plated onto LB agar plates with 200 μg/mL ERY. The constructed plasmid, pMZ4, was miniprepped from a single colony and confirmed by sequencing.

To use the pMZ4 plasmid to generate the Δ1245-WT strain, the helper plasmid pTRK669 was first electro-transformed into freshly prepared competent cells of *L. gasseri* ATCC 33323, then plated onto MRS agar plates with 7.5 μg/mL CHLOR and incubated anaerobically for 48 hours at 37°C. Clones containing pTRK669 (*L. gasseri* ATCC 33323/pTRK669) were verified by colony PCR screening (primers: pTRK699_F1 and pTRK699_R1). Next, pMZ4 was electro-transformed into freshly prepared competent cells of verified *L. gasseri* ATCC 33323/pTRK669, which were then plated onto MRS agar plates with 7.5 μg/mL CHLOR and 5 μg/mL ERY and incubated anaerobically for 48 hours at 37°C. ATCC 33323/pTRK669+pMZ4 clones were verified by colony PCR screening (primers: pTRK699_F1 and pTRK699_R1; pORI28F1 and pORI28R1). ATCC 33323/pTRK669+pMZ4 was then broth cultured under 7.5 μg/mL CHLOR and 5 μg/mL ERY selection at 37°C, then subcultured 3–5 times under 2.5 μg/mL ERY selection only at 42°C to cure the pTRK669 helper plasmid and select for pMZ4 single-crossover chromosomal integrants, then plated onto MRS agar plates with 5 μg/mL ERY to identify pMZ4 single-crossover chromosomal integrant clones. pMZ4-integration clones verified by colony PCR screening (using primers 1245-up and 1245-dwn) were cultured without antibiotic selection, then subcultured into MRS broth with 100 μg/mL 5-FU to counter-select for a second crossover event to produce the *upp* gene deletion mutant strain, then plated onto MRS media agar with 100 μg/mL 5-FU and incubated anaerobically for 48 hours at 37°C. Multiple subcultures were grown in parallel to increase chances of obtaining an *upp* gene deletion strain. Obtained colonies were PCR-screened (primers: 1245-up and 1245-dwn, amplicon lengths: 2.45kbp in WT and 1.29kbp in KO) and single-colony purified 1–2 times. Purified clones were verified by WGS to confirm deletion of *upp* by double homologous recombination. This *upp*-deleted (Δ1245-WT) strain (referred to in figures and main text as WT) was thus 5-FU resistant.

To construct the pMZ7 backbone vector for generating gene-specific knockouts, *lacZ* and the *L. acidophilus upp* gene (*Laupp*) were cloned into pORI28 using two-piece Gibson cloning. In brief, *lacZ* and *Laupp* were each amplified from pUC19 and genomic DNA extracted from *L. acidophilus* ATCC 4356, respectively, to produce PCR amplicons, *lacZ* and *Laupp* (primers for *lacZ*: pUC19lacZ-F and pUC19lacZ-R; primers for *LAupp*: upp-F and upp-R). Splicing by overlap-extension PCR was used to fuse *lacZ* (780 bp) and *LAupp* (759 bp), producing a single 1519-bp amplicon (primers: pUC19lacZ-F and upp-R), which was gel-purified. The fused *lacZ*-*LAupp* amplicon and pORI28 were each PCR-amplified separately (primers for pORI28: lacZupp_intF1 and lacZupp_intR1; primers for *lacZ*-*LAupp*: lacZupp_intF2 and lacZupp_intR2) and assembled using NEB Gibson Master Mix (NEB #E2611). 10 μL of the Gibson reaction was used to transform competent EC1000 cells, then plated onto LB agar plates with 200 μg/mL ERY. The constructed plasmid, pMZ7, was miniprepped from a single colony and confirmed by sequencing.

To generate pMZ7-derived vectors for generating gene-specific deletion mutants in *L. gasseri* Δ1245-WT, pMZ9 (containing homology arms for *ohyA9*, LGAS_1351) and pMZ10 (containing homology arms for *farE*, LGAS_1630) were constructed using three-piece Gibson cloning in EC1000 cells. For pMZ9 construction, pMZ7 was linearized by PCR amplification (primers: LGAS_1351F9 and LGAS_1351R9). A 600-bp in the upstream region of LGAS_1351 (primers: LGAS_1351F10 and LGAS_1351R10) and a 600-bp in the downstream region of LGAS1351 (primers: LGAS_1351F11 and LGAS_1351R11) were PCR-amplified. All amplicons products were combined via Gibson reaction to make pMZ9. For pMZ10 construction, pMZ7 was linearized by PCR amplification (primers: LGAS_1630F9 and LGAS_1630R9). A 628-bp in the upstream region of LGAS_1630 (primers: LGAS_1630F10 and LGAS_1630R10) and a 627-bp in the downstream region of LGAS1630 (primers: LGAS_1630F11 and LGAS_1630R11) were PCR-amplified. All amplicons products were combined via Gibson reaction to make pMZ10.

To generate gene deletion mutants in Δ1245-WT, pTRK669 was electro-transformed into freshly prepared competent cells of Δ1245-WT, then plated onto MRS agar plates with 7.5 μg/mL CHLOR and incubated anaerobically for 48 hours at 37°C. Clones containing pTRK669 (Δ1245-WT/pTRK669) were verified by colony PCR screening (primers: pTRK699_F1 and pTRK699_R1). Next, the pMZ7-derived vector containing homology arms for the gene of interest (pMZ9 or pMZ10) was electro-transformed into freshly prepared competent cells of Δ1245-WT/pTRK669, then plated onto MRS agar plates with 7.5 μg/mL CHLOR and 5 μg/mL ERY and incubated anaerobically for 48 hours at 37°C. Δ1245-WT/pTRK669+pMZ7-derived_vector clones (pMZ9 or pMZ10) were verified by colony PCR screening (primers for PTRK669: pTRK699_F1 and pTRK699_R1; primers for pMZ7-derived vector: pORI28F1 and pORI28R1). To select for pMZ7-derived_vector chromosomal integration clones, Δ1245-WT/pTRK669+pMZ7-derived_vector was subjected to the same protocol described above for generating pMZ4 single-crossover chromosomal integrant clones from ATCC 33323/pTRK669+pMZ4. Colonies were PCR-screened for pMZ7-derived_vector chromosomal integration (primers for LGAS_1351: S1351_F1 and S1351_R2; primers for LGAS_1630: S1630_F1 and S1630_R2). To isolate the desired gene deletion mutant, verified pMZ7-derived_vector-integration clones were subjected to the same protocol described above for generating the Δ1245-WT strain from the pMZ4-integration clones. Obtained colonies were PCR-screened with primers flanking the gene of interest, using primers S1351_F1 and S1351_R2 for LGAS_1351 (expected amplicon lengths: 2.0kb for WT and 1.2kb for *ΔohyA9*) and primers S1630_F1 and S1630_R2 for LGAS_1630 (expected amplicon lengths 3.9kb for WT and 1.2kb for *ΔfarE*; Figure S4L) and single-colony purified 1–2 times. Purified clones were verified by WGS to confirm deletion of the gene.

To construct expression vectors for genetic complementation, genes were cloned into pTRK892,^62^ an erythromycin resistant vector backbone containing a strong Ppgm promoter, using Gibson cloning. In brief, pTRK892 was linearized by PCR amplification such that the strong Ppgm promoter was retained and original gene insert, a mutated form of GusA, was excluded (primers: pTRK892_F1G and pTRK892_R1G). To construct pMZ12 (the *ohyA9* expression vector, also referred to a p*ohyA9*), *ohyA9* (LGAS_1351) was amplified from purified genomic DNA from *L. gasseri* ATCC 33323 (primers: 1351.FOR and 1351.REV) and combined with the linearized pTRK892 via a Gibson reaction. To construct pMZ13 (the *farE* expression vector, also referred to as p*farE*), *farE* (LGAS_1630) was amplified from purified genomic DNA from *L. gasseri* ATCC 33323 (primers: 1630G.FOR and 1630G.REV) and combined with the linearized pTRK892 via a Gibson reaction. Constructs were transformed into competent MC1061 and plated onto LB agar with 200 μg/mL ERY. Plasmids were miniprepped from a single colony and confirmed by sequencing. pMZ12 was electro-transformed into freshly prepared competent cells of *ΔohyA9* to make *ΔohyA9*/p*ohyA9*, and pMZ13 was electro-transformed into freshly prepared competent cells of *ΔfarE* to make *ΔfarE*/p*farE*. Transformed cells were plated onto MRS agar plates with 5 μg/mL ERY and incubated anaerobically for 48 hours at 37°C. Complementation was verified by colony PCR-screening (primers: Erm_F1 and Erm_R1, amplicon size 250 bp).

#### Plasmid construction and complementation in *Staphylococcus aureus* USA300 *ΔSaohyA*

All vectors and genetic mutants generated in *S. aureus* are noted in the key resources table and Table S4, respectively. To construct vectors for genetic complementation in the *ohyA*-knockout *S. aureus* USA300 strain (*ΔSaohyA*), His-tagged LCRIS_00661 and LCRIS_00558 genes with the appropriate restriction sites were ordered from Invitrogen and subcloned into a previously constructed *S. aureus* expression vector^49^ (pPJ480) using restriction enzymes NcoI and XhoI in TOP10 chemically competent *E. coli* (Invitrogen #C4040) to make pLCRIS_00661 and pLCRIS_00558. Constructed plasmids were verified by nanopore-based whole plasmid sequencing using Primordium Labs services. Plasmids were next laundered through *S. aureus* strain RN4220, which can accept plasmids propagated through *E. coli* due its inactivated restriction system,^98^^,^^99^ and then *S. aureus* RN4220-derived plasmids were purified and used to transform *ΔSaohyA*. Electroporation was used to transform all *S. aureus* strains. Briefly, 1.5–2 μg of DNA was incubated with the *S. aureus* strain on ice for 5 min, the mixture was then electroporated in pre-chilled 0.1 cm gap Gene Pulser Electroporation Cuvettes (Bio-rad Laboratories #1652089) with a Gene Pulser Xcell Electroporation System using the following conditions: 1.6 kV, 25 μF, and 200 Ω. Immediately after electroporation, cells were incubated for 2 hours in Brain Heart Infusion broth (BHI; BD Biosciences #DF0037) at 37°C with shaking. After incubation, cells were gently pelleted (300xg, 5 min at RT), plated onto BHI agar plates with 10 μg/mL CHLOR, and incubated overnight at 32°C.

#### Enzyme product characterization

In order to measure production of OhyA metabolites, strains *ΔSaohyA*/empty vector, *ΔSaohyA*/p*SaohyA*, *ΔSaohyA*/pLCRIS_00558, and *ΔSaohyA*/pLCRIS_00661 were grown to an OD600 of 0.5 in Tryptone broth containing 1% DMSO. OA or LOA was added to a final concentration of 20 μM, and the cultures were grown for 1 hour at 37°C with shaking. The cells were separated from media by centrifugation, and the medium was extracted by adding methanol to a final concentration of 80%. Extracts were centrifuged to pellet debris, and the supernatant was analyzed by LC-MS as described below to detect the presence of the *h*FAs.

Culture supernatants containing the fatty acid substrate and *h*FA product were analyzed with a Shimadzu Prominence UFLC attached to a QTrap 4500 equipped with a Turbo V ion source (Sciex). Samples were injected onto an XSelect® HSS C18, 2.5 μm, 3.0 × 150-mm column (Waters) at 45°C with a flow rate of 0.4 ml/min. Solvent A was water, and solvent B was acetonitrile. The HPLC program was as follows: starting solvent mixture of 60% B, 0–1 min isocratic with 60% B; 1–16 min linear gradient to 100% B; 16–21 min isocratic with 100% B; 21–23 min linear gradient to 0% B; and 23–28 min isocratic with 0% B. The Sciex QTrap 4500 was operated in the negative mode, and the ion source parameters were: ion spray voltage, −4500 V; curtain gas, 30 psi; temperature, 320°C; collision gas, medium; ion source gas 1, 20 psi; ion source gas 2, 35 psi; and declustering potential, −35 V. The system was controlled by Analyst® software (Sciex).

The Sciex QTrap 4500 mass spectrometer was operated in the negative mode using the product scan to determine the position of the hydroxyl group by direct injection. The source parameters were: ion spray voltage, −4500 V; curtain gas, 15 psi; temperature, 250°C; collision gas, high; ion source gas 1, 15 psi; ion source gas 2, 20 psi; declustering potential, −25 V; and collision energy, −35 V. The system was controlled by Analyst® software (Sciex).

#### Targeted lipidomics for the detection of *h*FAs in cervicovaginal lavage samples

Picolylamide derivatization was used to sensitively and accurately detect *h*FA abundance in CVL supernatant samples using the unique ions generated from breakage at the hydroxyl group position.^56^^,^^58^ 250 μL of each human CVL sample was added to 750 μL of methanol containing 200 nM ^13^C_18_-OA (Cambridge Isotope Laboratories, Inc. #CLM-460-PK), incubated on ice for 15 min, and centrifuged at 4,000 rpm for 10 min. Supernatant was removed to a new tube and dried in a speed-vac overnight. 500 μL of oxalyl-chloride was added to the dried samples and incubated at 65°C for 15 min. After drying the samples under N_2_ gas, 500 μL of 1% 3-picolylamine in acetonitrile was added and incubated at room temperature for 15 min. After drying the samples under N_2_ gas, the samples were resuspended in 100 μL of ethanol for analysis.

Picolylamide-*h*FA were analyzed using a Shimadzu Prominence UFLC attached to a QTrap 4500 equipped with a Turbo V ion source (Sciex). Samples (5 μL) were injected onto an XSelect HSS C18, 2.5 μm, 3.0 x 150 mm column (Waters) at 45°C with a flow rate of 0.4 mL/min. Solvent A was 0.1% formic acid in water, and solvent B was acetonitrile with 0.1% formic acid. The HPLC program was the following: starting solvent mixture of 70% A/30% B; 0 to 5 min, isocratic with 30% B; 5 to 15 min, linear gradient to 100% B; 15 to 23 min, isocratic with 100% B; 23 to 25 min, linear gradient to 30% B; 25 to 30 min, isocratic with 30% B. The QTrap 4500 was operated in the positive mode, and the ion source parameters for the picolylamide-*h*FA multiple reaction monitoring (MRM) parameters were: ion spray voltage, 5,500 V; curtain gas, 15 psi; temperature, 300°C; ion source gas 1, 15 psi; ion source gas 2, 20 psi; declustering potential, 25 V, and a collision energy, 40 V. MRM masses (Q1/Q3) were: picolylamide-*h*18:0, 391.1/109.0; picolylamide-*h*18:1, 389.1/109.0; and picolylamide-^13^C_18_-OA, 391.1/109.0. The system was controlled by the Analyst software (Sciex) and analyzed with MultiQuant™ 3.0.2 software (Sciex). The relative concentration of each *h*FA was calculated based on the known amount of ^13^C_18_-OA spiked into the sample at the beginning of the sample preparation.

#### Targeted lipidomics for the detection of PG metabolites in cell pellets

Lipids were extracted from bacterial cell pellets using the Bligh and Dyer method.^100^ In brief, cell pellets were homogenized with a 1:2 mixture of chloroform:methanol with bead beating. The mixture was then filtered through Whatman No. 1 filter paper (Millipore Sigma #WHA1001090), and the filtrate was allowed to separate into two layers, an alcohol and chloroform layer. The alcohol layer was removed and the remaining chloroform layer contained the lipid extract. Lipid extracts were resuspended in chloroform/methanol (1:1). PG was analyzed using a Shimadzu Prominence UFLC attached to a QTrap 4500 equipped with a Turbo V ion source (Sciex). Samples were injected onto an Acquity UPLC BEH HILIC, 1.7 um, 2.1 x 150 mm column (Waters) at 45°C with a flow rate of 0.2 ml/min. Solvent A was acetonitrile, and solvent B was 15 mM ammonium formate, pH 3. The HPLC program was the following: starting solvent mixture of 96% A/4% B; 0 to 2 min, isocratic with 4% B; 2 to 20 min, linear gradient to 80% B; 20 to 23 min, isocratic with 80% B; 23 to 25 min, linear gradient to 4% B; 25 to 30 min, isocratic with 4% B. The QTrap 4500 was operated in the Q1 negative mode. The ion source parameters for Q1 were as follows: ion spray voltage, –4,500 V; curtain gas, 25 psi; temperature, 350°C; ion source gas 1, 40 psi; ion source gas 2, 60 psi; and declustering potential, –40 V. The system was controlled by the Analyst software (Sciex). The sum of the areas under each peak in the mass spectra were calculated, using LipidView software (Sciex).

#### Untargeted metabolite profiling and isotopic tracing methods and analysis

C18-neg: Reversed-phase C18 chromatography coupled with negative ion mode MS detection was used to measure fatty acids with an LC-MS system consisting of a Shimadzu Nexera X2 U-HPLC (Shimadzu Corp.) coupled to a Q- Exactive orbitrap mass spectrometer (Thermo Fisher Scientific). Media samples (30 μL) were extracted for analyses using 90 μL of methanol containing 50ng/mL 15-methyl PGE1, 15-methyl PGA2, 15-methyl PGE2 (Cayman Chemical Co.) as internal standards. Cell pellet samples (30 μL) were extracted for analyses using 90 μL of methanol containing 50ng/mL 15-methyl PGE1, 15-methyl PGA2, 15-methyl PGE2 (Cayman Chemical Co.) as internal standards. The cell pellets were homogenized using the QIAGEN TissueLyser II with 3mm Tungsten beads for 4 min at a frequency of 20 Hz. Samples were centrifuged (10 min, 15000xg at 4°C). After centrifugation, supernatants (2 μL) were injected directly onto a 150 x 2.1 mm, 1.8 μm ACQUITY HSS T3 C18 column (Waters). The column was eluted isocratically with 80% mobile phase A (0.01% formic acid in water) for 3 min followed by a linear gradient to 100% mobile phase B (0.01% acetic acid in acetonitrile) over 12 min. MS analyses were carried out using electrospray ionization in the positive ion mode using full scan analysis over 70–850 m/z at 70,000 resolution and 3 Hz data acquisition rate. Other MS settings were: sheath gas 45, in source CID 5 eV, sweep gas 10, spray voltage –3.5 kV, capillary temperature 320°C, S-lens RF 60, probe heater temperature 300°C, microscans 1, automatic gain control target 1e6, and maximum ion time 250 ms. Raw data were processed using TraceFinder software (Thermo Fisher Scientific) for targeted peak integration and manual review of a subset of identified metabolites and using Progenesis QI (Nonlinear Dynamics) for peak detection and integration of both metabolites of known identity and unknowns. Reference standards used for the identification of hydroxystearic acids used are: DL-alpha-hydroxystearic acid (H9631, Sigma) and 10-Hydroxyoctadecanoic acid (A125712, Ambeed).

C8-pos: Reversed-phase C8 chromatography/positive ion mode MS detection was used to measure lipids. Analyses of polar and non-polar lipids were conducted using an LC-MS system comprising a Shimadzu Nexera X2 U-HPLC (Shimadzu Corp.) coupled to an Exactive Plus orbitrap mass spectrometer (Thermo Fisher Scientific). Media samples (10 μL) were extracted for lipid analyses using 190 μL of isopropanol containing 1,2-didodecanoyl-sn-glycero-3-phosphocholine (Avanti Polar Lipids) as an internal standard. Cell pellets (∼30ul) were extracted for lipid analysis using 570ul isopropanol containing 1,2-didodecanoyl-sn-glycero-3-phosphocholine (Avanti Polar Lipids) as an internal standard. The cell pellets were homogenized using the QIAGEN TissueLyser II with 3mm Tungsten beads for 4 min at a frequency of 20Hz. The media and cell pellets were centrifuged (10 min, 9,000xg at 4°C), and were injected directly onto a 100 x 2.1 mm, 1.7 μm ACQUITY BEH C8 column (Waters). The column was eluted isocratically with 80% mobile phase A (95:5:0.1 vol/vol/vol 10mM ammonium acetate/methanol/formic acid) for 1 minute followed by a linear gradient to 80% mobile-phase B (99.9:0.1 vol/vol methanol/formic acid) over 2 min, a linear gradient to 100% mobile phase B over 7 min, then 3 min at 100% mobile-phase B. MS analyses were carried out using electrospray ionization in the positive ion mode using full scan analysis over 200–1100 m/z at 70,000 resolution and 3 Hz data acquisition rate. Other MS settings were: sheath gas 50, in source CID 5 eV, sweep gas 5, spray voltage 3 kV, capillary temperature 300°C, S-lens RF 60, heater temperature 300°C, microscans 1, automatic gain control target 1e6, and maximum ion time 100 ms. Raw data were processed using TraceFinder software (Thermo Fisher Scientific) for targeted peak integration and manual review of a subset of identified lipids and using Progenesis QI (Nonlinear Dynamics) for peak detection and integration of both lipids of known identity and unknowns. Lipid identities were determined based on comparison to reference plasma extracts and are denoted by total number of carbons in the lipid acyl chain(s) and total number of double bonds in the lipid acyl chain(s).

HILIC-neg: HILIC (hydrophilic interaction chromatography) analyses of water soluble central metabolites metabolites in the negative ionization mode (HILIC-neg) were conducted using an LC-MS system comprised of an Shimadzu Nexera X2 U-HPLC (Shimadzu Corp.; Marlborough, MA) coupled to a Q Exactive Plus hybrid quadrupole orbitrap mass spectrometer (Thermo Fisher Scientific; Waltham, MA). Media samples (30 μL) were extracted with the addition of four volumes of 80% methanol containing inosine-15N4, thymine-d4 and glycocholate-d4 internal standards (Cambridge Isotope Laboratories). Cell pellets (∼30ul) were extracted with the addition of four volumes of 80% methanol containing inosine-15N4, thymine-d4 and glycocholate-d4 internal standards (Cambridge Isotope Laboratories. The cell pellets were homogenized using the QIAGEN TissueLyser II with 3mm Tungsten beads for 4 min at a frequency of 20 Hz. The samples were incubated at 4°C for one hour centrifuged (10 min, 9,000xg at 4°C), and the supernatants were injected directly onto a 150 x 2.0 mm Luna NH2 column (Phenomenex). The column was eluted at a flow rate of 400 μL/min with initial conditions of 10% mobile phase A (20 mM ammonium acetate and 20 mM ammonium hydroxide in water) and 90% mobile phase B (10 mM ammonium hydroxide in 75:25 v/v acetonitrile/methanol) followed by a 10 min linear gradient to 100% mobile phase A. MS analysis was with electrospray ionization (ESI) in the negative ion mode with the following parameters with full scan analysis over m/z 70–750 at 70,000 resolution and 3 Hz data acquisition rate. Raw data was processed using TraceFinder (Thermo Fisher Scientific; Waltham, MA) and Progenesis QI (Nonlinear Dynamics; Newcastle upon Tyne, UK). Metabolite identities were confirmed using authentic reference standards or reference samples.

#### Nucleic acid extraction for 16S rRNA gene sequencing

For vaginal swab samples, total nucleic acids were extracted from the swab samples using the phenol-chloroform method, which includes a bead beating process to disrupt bacteria, as previously described.^13^^,^^101^ Briefly, swabs were thawed on ice, transferred into a solution consisting of phenol:chloroform:isoamyl alcohol (PCI, 25:24:1, pH 7.9, Ambion) and 20% sodium dodecyl sulfate in Tris-EDTA buffer with sterile 0.1 mm glass beads (BioSpec Products #11079101), vigorously rubbed against the walls of the tube to dislodge microbial material, and then incubated on ice for 5–10 min. Swabs were then removed by pressing the swab against the side of the tube using a sterile pipette tip as being lifted out to squeeze out excess fluid. Samples were homogenized using a bead beater for 2 min at 4°C, and then centrifuged at 6,800×g for 3 min at 4°C. The aqueous phase was transferred to a clean tube with equal volume of PCI solution, vortexed, and centrifuged again at 16,000×g for 5 min at 4°C. The aqueous phase was transferred to a second clean tube, precipitated using 0.8 volume of −20°C isopropanol with 0.08 volume (relative to initial sample) 3 M sodium acetate at pH 5.5, inverted to mix, and incubated overnight at −20°C. Samples were then centrifuged for 30 min at 21,100×g at 4°C, washed in 0.5 ml 100% ethanol and centrifuged for 15 min at 21,100×g at 4°C. The ethanol supernatant was discarded while keeping the pellet, which was allowed to air-dry and then resuspended in 20μl molecular-grade Tris-EDTA buffer. Genomic DNA from mock communities cultured in vitro was extracted using a plate-based adaptation of the above protocol including a bead beating process combined with phenol–chloroform isolation with QIAamp 96 DNA QIAcube HT kit (Qiagen Beverly LLC #51331) protocols.^36^

#### 16S rRNA gene sequencing for vaginal swab and mock community samples

Bacterial microbiota composition from vaginal swabs collected from FRESH study participants and compositions of defined bacterial mock community experiments were determined using Illumina-based amplicon sequencing of the V4 region of the bacterial 16S rRNA gene. Standard PCR-amplification protocols were used to amplify the V4 region of the bacterial 16S rRNA gene.^13^^,^^101^^,^^102^ Briefly, samples were amplified using 0.5 units of Q5 high-fidelity DNA polymerase (NEB #M0491S) in 25 μl reaction with 1X Q5 reaction buffer, 0.2 mM deoxyribonucleotide triphosphate mix, 200 pM 515F primer (5’- AATGATACGGCGACCACCGAGACGTACGTACGGTGTGCCAGCMGCCGCGGTAA-3’, the underlined sequence representing the complementary region to the bacterial 16S rRNA gene; IDT) and 200 pM barcoded 806R primer (5’-CAAGCAGAAGACGGCATACGAGATXXXXXXXXXXXXAGTCAGTCAGCCGGACTACHVGGGTWTCTAAT-3’, the underlined sequence representing the complementary region to the bacterial 16S rRNA gene and the X characters representing the barcode position; IDT) in PCR-clean water. For each prepared barcode master mix, a water-template negative control reaction was performed in parallel. Blank extraction and amplification controls were additionally performed using unique barcoded primers in sequencing libraries. For FGT microbiota profiling from vaginal swab samples, DNA was amplified in triplicate reactions and then triplicates were combined before library pooling to minimize stochastic amplification biases. For defined bacterial mock community experiments, DNA was amplified in a single reaction per replicate culture. Amplification was performed at 98°C for 30 s, followed by 30 cycles of 98°C for 10 s, 60°C for 30 s, and 72°C for 20 s, with a final 2 min extension at 72°C.

PCR products from all samples and the matched water-template control were visualized via agarose gel electrophoresis to confirm successful target amplification and absence of background amplification. Gel band strength was used to semi-quantitatively estimate relative amplicon concentrations for library pooling. To prepare the sequencing libraries, 3–20 μl of individual PCR products (adjusted on the basis of estimated relative amplicon concentration) were combined into 100 μl subpools and purified using an UltraClean 96 PCR cleanup kit (Qiagen Beverly LLC #12596-4). Despite not producing visible PCR bands, blank extractions, water-template and (for in vitro experiments) blank media controls were included in the sequencing libraries for additional quality control verification. Concentrations of the subpools were quantified using a Nanodrop 2000 and then pooled at equal molar concentrations to assemble the final library. Following standard Illumina protocols, the pooled library was diluted and supplemented with 10% PhiX, and then single-end sequenced on an Illumina MiSeq using a v2 300-cycle sequencing kit with addition of custom Earth Microbiome Project sequencing primers (read 1 sequencing primer: 5’-ACGTACGTACGGTGTGCCAGCMGCCGCGGTAA-3’; read 2 sequencing primer: 5’-ACGTACGTACCCGGACTACHVGGGTWTCTAAT-3’; index sequencing primer: 5’-ATTAGAWACCCBDGTAGTCCGGCTGACTGACT-3’; IDT).^102^ Vaginal swab samples were sequenced in two different sequencing runs. Samples from each bacterial mock community experiment were sequenced in the same sequencing run to minimize variation.

#### Analysis of 16S rRNA gene sequencing results

QIIME I v1.9.188^103^ was used to demultiplex Illumina MiSeq bacterial 16S rRNA gene sequence data. QIIME 1-formatted mapping files were used and validated using validate_mapping_file.py, sequences were demultiplexed using split_libraries_fastq.py with parameter store_demultiplexed_fastq with no quality filtering or trimming, and demultiplexed sequences were organized into individual fastq files using split_sequence_file_on_sample_ids.py.^101^ Dada2 v1.6.089^104^ in R was used to filter and trim reads at positions 10 (left) and 230 (right) using the filterAndTrim function with parameters truncQ=11, MaxEE=2 and MaxN=0. Then, sequences were inferred and initial taxonomy assigned using the dada2 assignTaxonomy function, employing the Ribosomal Database Project training database rdp_train_set_16.fa.gz (https://www.mothur.org/wiki/RDP_ reference_files). Data from the two vaginal swab sequencing runs were analyzed separately in dada2, then the resulting denoised data were combined for further analysis. Taxonomic assignments were refined and extended via manual review. Phlyoseq v1.30.090^105^ in R was used to analyze and process the denoised dada2 results with final taxonomic assignment and custom R scripts. Final analysis and visualization of results were performed in python using jupyter notebooks.

For 16S rRNA gene-based microbiome profiling of clinical samples, microbial communities were classified into 4 cervicotypes (CTs) as previously defined^13^ in a non-overlapping subset of participants from the FRESH cohort: CT1 includes communities with >50% relative abundance of non-*iners Lactobacillus* species (which consists almost entirely of *L. crispatus* in this population); CT2 consists of communities in which *L. iners* is the most dominant taxon; CT3 consists of communities in which the genus *Gardnerella* is the most dominant taxon; and CT4 consists of communities dominated by other species, typically featuring high abundance of one or more *Prevotella* species. ASVs that could not be defined to the level of taxonomic class were pruned from the dataset. Taxonomically defined ASVs were collapsed at the species or genus level as indicated for further visualization and statistical analyses. Sequencing-based analysis of FGT microbiota composition from FRESH cohort swab samples, taxonomy assignment, and cervicotype assignments was performed blinded to participants’ corresponding Nugent scores and cervicovaginal *h*FA concentrations.

For 16S rRNA gene sequence analysis for bacterial mock community experiments, sequences were generated, processed, and annotated as described above. For each community replicate, relative abundances of each experimental strain were determined and the ratios of non-*iners* FGT *Lactobacillus* species read counts to the sum of the read counts of all of the other experimental strains were determined to quantify non-*iners* FGT *Lactobacillus* species enrichment for each condition. Significance of between-group differences for each mixture was determined by one-way ANOVA, and significance of pairwise comparisons was calculated using Tukey’s test, with statistical values for all pairwise conditions reported in Table S3.

#### Genomic analyses of FGT *Lactobacillus* and other species

All genomes of experimentally tested strains were obtained from RefSeq or Genbank. We utilized the extensive genome collection of FGT *Lactobacillus* species reported by Bloom et al. (including isolate genomes and metagenome-assembled genomes)^36^ and the type strain genome sequences of species in the family Lactobacillaceae reported in Zheng et al.^55^ to identify presence/absence profiles of genes of interest across FGT *Lactobacillus* and Lactobacillaceae species. All genomes were downloaded from NCBI RefSeq or GenBank. Gene prediction for all genomes was performed using Prodigal, and gene functions were predicted using eggNOG 5.0^106^ employing eggNOG-mapper v2.1.9.^107^ Custom Python scripts were used to parse the eggNOG outputs to identify the presence of genes or gene functions of interest in each genome. We constructed a gene presence and absence map for all genes and gene functions of interest across all genomes. For each gene of interest, MUSCLE v5.1^108^ was used for multiple sequence alignment of representative orthologs, ModelTest-NG^109^ was used to select the optimal substitution model, and RAxML-NG^110^ used for tree construction employed via raxmlGUI 2.0^111^ to map their phylogenetic relationships. For the construction of species phylogeny, core ribosomal genes present in all Lactobacillaceae genomes were aligned using MUSCLE v5.1^108^ and FastTree v2.1^112^ was used to construct the Lactobacillaceae species tree. Individual protein trees were constructed using ortholog sequences from each species that were representative of the majority of that protein’s sequences found in the respective orthologous group in that species to ensure robustness of the phylogenetic reconstruction. Tree and corresponding metadata visualization was done using Interactive Tree Of Life (iTOL) v5.^113^

### Quantification and statistical analysis

For bacterial growth assays, figures depict representative results from 1 of ≥2 independent experiments prepared with distinct batches of media and bacterial input inocula. Growth data collection and analysis was not blinded to the conditions of the experiments. For bulk RNA-sequencing samples, all conditions were performed in triplicate cultures performed in parallel, but with each individual culture inoculum coming from a unique bacterial colony. For mass spectrometry experiments, each condition included results from duplicate or triplicate replicate cultures prepared from a single, common starter culture. For defined *in vitro* bacterial community assays, all conditions were performed with six replicate cultures seeded from the same input inoculum and grown in parallel.

Data analysis, statistics, and visualization were performed using custom scripts written in python v3.9 using Jupyter Notebook v6.5.2 or R v.3.6.3. R packages used for analyses and plotting include seqinr v.4.2.5, tidyverse, v.1.3.1, knitr v.1.33, ggpubr v.0.4.0, DescTools v.0.99.41, gtools v.3.8.2, gridExtra v.2.3, cowplot v.1.1.1, scales v.1.1.1, grid v.3.6.3, broom v.0.7.6, e1071 v.1.7.6, and table1 v.1.4. Python packages used for analyses and plotting include biopython v1.79, matplotlib v3.7.1, numpy v1.22.3, pandas v1.5.1, scikit-bio v0.5.8, scipy v1.9.3, seaborn v0.11.2, statannot v0.2.3, and statsmodels v0.13.2. All *p* values are two-sided with statistical significance defined at α=0.05, unless otherwise indicated.

## Supplemental information

Table S1. FGT bacterial isolates, related to STAR Methods

Table S2. Human specimens with associated targeted lipidomic and microbiome data and sequence accession numbers, related to Figure 4

Table S3. Statistical values, related to Figures 4, 7, and S7

Table S4. Primers, related to STAR Methods
